# Harnessing the Biomimetic Effect of Macromolecular Crowding in the Cell-Derived Model of Clubfoot Fibrosis

**DOI:** 10.1021/acs.biomac.4c00653

**Published:** 2024-08-30

**Authors:** Martina Doubková, Jarmila Knitlová, David Vondrášek, Adam Eckhardt, Tomáš Novotný, Martin Ošt’ádal, Elena Filová, Lucie Bačáková

**Affiliations:** † Laboratory of Biomaterials and Tissue Engineering, Institute of Physiology of the Czech Academy of Sciences, Videnska 1083, 142 00 Prague 4, Czech Republic; ‡ Second Faculty of Medicine, Charles University, V Uvalu 84, 150 06 Prague 5, Czech Republic; § Faculty of Science, Charles University, Albertov 6, 128 00 Prague 2, Czech Republic; ∥ Laboratory of Biomathematics, Institute of Physiology of the Czech Academy of Sciences, Videnska 1083, 142 00 Prague 4, Czech Republic; ⊥ Laboratory of Translational Metabolism, Institute of Physiology of the Czech Academy of Sciences, Videnska 1083, 142 00 Prague 4, Czech Republic; # Department of Orthopaedics, Masaryk Hospital, Socialni Pece 3316/12A, 401 13 Usti nad Labem, Czech Republic; ∇ Department of Histology and Embryology, Second Faculty of Medicine, Charles University, V Uvalu 84, 150 06 Prague 5, Czech Republic; ○ Department of Orthopaedics, University Hospital Bulovka, Charles University, Budinova 67/2, 180 81 Prague 8, Czech Republic

## Abstract

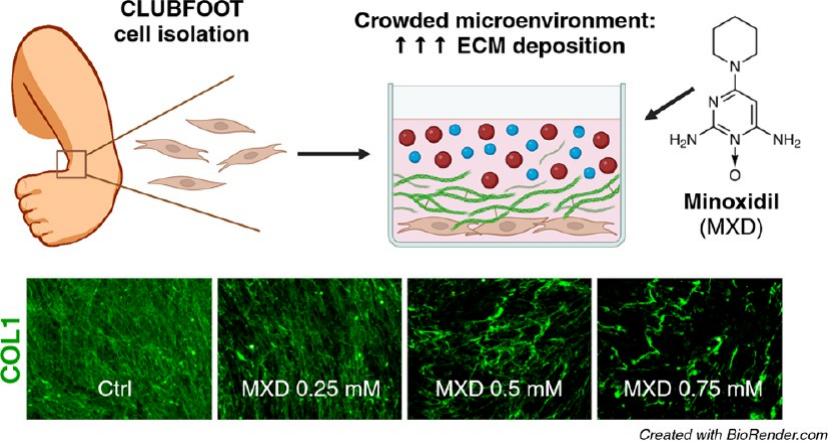

Fibrotic changes in pediatric clubfoot provide an opportunity to improve corrective therapy and prevent relapses with targeted drugs. This study defines the parameters of clubfoot fibrosis and presents a unique analysis of a simple pseudo-3D *in vitro* model for disease-specific high-throughput drug screening experiments. The model combines clubfoot-derived fibroblasts with a biomimetic cultivation environment induced by the water-soluble polymers Ficoll and Polyvinylpyrrolidone, utilizing the principle of macromolecular crowding. We achieved higher conversion of soluble collagen into insoluble collagen, accelerated formation of the extracellular matrix layer and upregulated fibrosis-related genes in the mixed Ficoll environment. To test the model, we evaluated the effect of a potential antifibrotic drug, minoxidil, emphasizing collagen content and cross-linking. While the model amplified overall collagen deposition, minoxidil effectively blocked the expression of lysyl hydroxylases, which are responsible for the increased occurrence of specific collagen cross-linking in various fibrotic tissues. This limited the formation of collagen cross-link in both the model and control environments. Our findings provide a tool for expanding preclinical research for clubfoot and similar fibroproliferative conditions.

## Introduction

Idiopathic clubfoot (*Talipes equinovarus*) is a common congenital lower limb deformity with a complex multifactorial etiology that is present in 1–2 in 1000 newborns worldwide.^1^ This defect comprises fixation of the foot in plantar flexion with inward rotation of the sole, which leads to the deformation of soft and hard tissues,^2,3^ resulting in moderate to severe mobility impairment. The stiffness of the deformed tissues poses a challenge for the main corrective methods of systematic serial manipulation, casting, and bracing.^4^ The Ponseti corrective method usually achieves excellent results, provided that the regimen is followed correctly. Unfortunately, approximately 30% of patients experience a relapse within 5 years after starting treatment, mainly due to patient and parental noncompliance.^5,6^ Patients must therefore repeatedly undergo the burden of corrective treatment with a decreasing chance of success or a surgical release may even be considered.^7^ When relapsed clubfoot is resistant to the main corrective treatment method, further treatment could be attempted with an adjunctive pharmaceutical approach. For example, the application of botulinum toxin A injections was proposed, but it failed to demonstrate an adequate positive effect on important aspects of the standard treatment and failed to prevent relapses of isolated idiopathic clubfoot during clinical trials.^8,9^ However, this neurotoxin has proved to be effective in the treatment of spastic clubfoot,^10^ where the contracture had different underlying causes.

The unclear etiology provides challenges in research on potential adjuvant or alternative treatment, but previous analyses of tissue biopsies of prenatal^2,11^ and relapsed idiopathic clubfeet^12−15^ suggest the presence of fibrotic changes localized in the stiff, contracted tissue on the medial side (CF-M side). More recent reports on the differences between the medial side tissue and the noncontracted lateral side (CF-L side) tissue have come primarily from work carried out by our research group, and are in agreement with earlier studies. The affected medial side tissue exhibits increased amounts of collagen type I, III, V, and VI,^16−18^ fibronectin, tenascin-C,^16,17,19^ transforming growth factor-β-induced protein (TGF-βip),^16,17^ transforming growth factor-β (TGF-β), smooth muscle α-actin,^19^ increased collagen cross-linking,^18^ and expression of cross-linking enzymes lysyl oxidase and lysyl oxidase-like 2.^19^ In addition, quantitative mechanical analysis of the clubfoot tissue has suggested relative stiffness of the medial side connective tissue compared to the lateral side.^20^ Together, these indications make the medial side a possible candidate for targeted treatment.

The accumulation of extracellular matrix (ECM) proteins, especially fibrillar collagens, and growth factors regulating cell behavior to drive the deposition of ECM are typical in connective tissue diseases such as Dupuytren’s contracture, Peyronie’s disease, hypertrophic scars and keloids (for a review, see Siani et al.^21^). The underlying process in these conditions is fibrosis. Only a small number of studies have approached this problem in clubfoot by suggesting the targeted application of antifibrotic agents. Researchers have suggested influencing the tissue properties through the downregulation of growth factors TGF-β and platelet-derived growth factor (PDGF)^14^ or β-catenin,^15^ which is part of a signaling pathway in crosstalk with TGF-β signaling. In contrast, our research group has focused on reducing cross-link formation in the produced ECM, which contributes to tissue stiffening by inhibiting enzymes acting in post-translational modifications of collagen, i.e., lysyl hydroxylases^22^ and lysyl oxidase.^18^ In addition to standard cell culture, we have employed floating collagen gels with embedded cells to simulate the studied antifibrotic drug effects on tissue-like contraction exerted by the traction forces of clubfoot-derived cells.^18,22^ However, the studies performed by other groups have been conducted entirely in conventional two-dimensional (2D) cell cultures.

Fibroblasts and other cells *in vivo* produce and are surrounded by their specific ECM microenvironment, which profoundly affects their phenotype and function. Tissue engineering focuses constantly on modulating various physical properties of the microenvironment (such as its topography or stiffness) and the chemical composition of the environment, which provide important cues for cell behavior. Especially in the investigation of fibrotic conditions, the dilute environment of a standard *in vitro* monolayer cell culture is not ideal for the deposition of ECM. This is primarily due to impaired collagen deposition as a result of the slow enzymatic conversion of procollagen to collagen.^23^ The phenomenon of macromolecular crowding (MMC) can be conveniently used to mimic the dense physiological environment, where the concentration of macromolecules is noticeably higher. The addition of specific inert macromolecules into the culture media induces an effect of excluded volume, limiting the space that can be accessed by other molecules, slowing their diffusion and facilitating enhanced ECM deposition *in vitro*.^23,24^ MMC has been observed to affect the aggregation and interaction of many ECM molecules, accelerating and increasing their deposition, affecting stem cell differentiation, affecting the regulation of gene expression, promoting cell signaling and increasing enzyme activity (for a comprehensive review, see Tsiapalis *et* Zeugolis^25^).

The concept of MMC in cell culture is not entirely new,^26,27^ but interest in its research applications has gradually increased in recent years, particularly in the field of tissue engineering and drug discovery.^24,28−35^ Recent studies have utilized MMC to analyze the effects of antifibrotic agents with or without simultaneous stimulation of cells by pro-fibrotic growth factors.^29,32,36,37^ MMCs have been successfully applied to cultures of mesenchymal stem cells,^30,38−41^ lung fibroblasts,^29,32,35^ dermal fibroblasts,^33,38,42−44^ fibroblasts from hypertrophic scars,^28^ tenocytes,^45,46^ and fibroblasts from the cornea.^31^ The most commonly used macromolecular crowders include Dextran, Dextran sulfate, Ficoll, Carrageenan, and Polyvinylpyrrolidone.

*In vitro* cell culture with the application of MMC allows the creation of a biomimetic environment mimicking the properties of the extracellular environment *in vivo*. The behavior of cells isolated from clubfoot in the MMC environment has not yet been investigated. Although there have been studies aiming to establish an animal model of clubfoot,^47,48^ no suitable *in vivo* model is yet available. It is therefore desirable to improve the *in vitro* experimental conditions for performing relevant analyses when carrying out research on adjuvant drug treatment.

The present study aimed to investigate the effect of macromolecular crowding (MMC) on primary fibroblast cell cultures isolated from the tissues of patients after surgery for relapsed idiopathic clubfoot. In the first part of the study, we aimed to develop an *in vitro* model based on clubfoot-derived cells cultured under MMC conditions that emulates the fibrotic environment in clubfoot, emphasizing collagen synthesis and deposition. In the second part of the study, we aimed to explore the environment induced by MMC in an antifibrotic drug screening setup with minoxidil, an inhibitor of lysyl hydroxylases.^49^

Lysyl hydroxylases (more specifically isoform 2; LH2) are the enzymes responsible for the increased occurrence of the specific collagen cross-linking (pyridinoline cross-links) observed in various fibrotic tissues, including hypertrophic scars and Dupuytren’s contracture.^50−52^ Inhibition of lysyl hydroxylase leads to a change in the type and the amount of cross-linking between collagen type I fibers, resulting in a less stiff and more easily degradable ECM.^53^ These properties of minoxidil could reduce tissue stiffness and contraction, making it a good candidate for developing potential adjuvant pharmacological therapy to accompany the standard treatment for relapsed clubfoot.

We have selected the two most commonly used crowders (Ficol70/Ficol400 mix and Polyvinylpyrrolidone) and for the first time we have compared the effect of a wide range of their concentrations (FVO 4–54% in culture medium) simulating different physiological environments^38^ on cell cultures of clubfoot-derived fibroblasts (CF-M, CF-L). Normal human fibroblasts (NHDFs) were used as a control. We evaluated the proliferation, viability, and collagen production of the three cell types, with regard to the morphology and the maturation of the deposited collagen fibers. In addition, we examined the effect of the culture medium with the best-performing MMC environment on the gene expression of selected extracellular matrix proteins and enzymes involved in post-translational modifications of collagen. To validate the model pro-fibrotic conditions, we compared the data with the analyses of clubfoot tissue. We also monitored the phases of collagen deposition in the form of soluble and insoluble fractions in the extracellular matrix (ECM) and the amount of pyridinoline and deoxypyridinoline cross-links in ECM cell layers. Finally, we tested the performance of minoxidil, an inhibitor of lysyl hydroxylases, in the established MMC environment in separate experiments.

## Methods

### Biological Samples and Cell Isolation

The primary fibroblast cell cultures used in this study were isolated from tissue samples of 7 patients (5 males, 2 females; median age 4.3 years, SD = 2.4; Table S1, Supporting Information Section 1) treated for idiopathic congenital *talipes equinovarus* (ICTEV; further referred to as clubfoot, CF). All of these patients had previously been treated using the Ponseti method. Two tissue samples were harvested from the patients during surgery on relapsed clubfoot after unsuccessful Ponseti treatment. Samples from the medial side of the foot (M-side; CF-M) represent contracted fibrotic tissue localized between the medial malleolus, the *sustentaculum tali* and the navicular bone. Samples from the lateral side of the foot (L-side; CF-L) represent noncontracted tissue from the surface of the calcaneocuboid joint. All tissue samples were processed within 1 h after surgical excision by mincing and enzymatic digestion to isolate the primary cell cultures. The process of isolation and characterization of the obtained cell culture was the same as we described in our previous study. Briefly, the cell cultures comprised approximately 96% fibroblasts, less than 3% myofibroblasts, less than 3% fibrocytes, less than 0.1% of vascular muscle cells, and no endothelial cells.^22^ Cell passages 1 to 3 were used in the experiments.

The tissue samples embedded in paraffin used for histological staining presented here were collected previously for the study of Novotny et al.^19^ and are described in the appropriate section.

The research was carried out under the Declaration of Helsinki (1964) and with the approval of the ethics committee of the participating institutions (University Hospital Bulovka, Ev. No. 1.6.2021/10070/EK-Z, June 2021; Institute of Physiology of the Czech Academy of Sciences, project No. NU22-10-00072, June 2021). Parents or legally designated representatives of all patients provided their written informed consent to participate. This study is analytical, prospective, level of evidence IIb.

### Reagents Used to Induce an MMC-Based Biomimetic Cell Culture Microenvironment

The reagents used to create an experimental cell culture environment, and their final concentrations, are summarized in this section. Neutral Ficoll and Polyvinilpyrrodilone were selected to induce macromolecular crowding in the cell culture medium. The final concentrations of the compounds were scaled to mimic the molecular crowding (MMC) of the physiological environments according to the % fractional volume occupancy (FVO, v/v) occupied by them in the culture medium, as referenced by Rashid et al.^38^ Ficoll PM 70 kDa (F2878, Sigma-Aldrich, USA) and Ficoll PM 400 kDa (F4375, Sigma-Aldrich, USA) were used to create a model of mixed crowding (Ficoll mix) at FVO 4, 9, 18, 36, and 54%. Polyvinylpyrrolidone 40 kDa (PVP40, Sigma-Aldrich, USA) was used at FVO 9, 18, 36, and 54% (Table 1).

**Table 1:** Groups of Macromolecular Crowders (MMC) Diluted in Media for the Experiments

	Ficoll mix		
% FVO	Ficoll 70 (mg/mL)	Ficoll 400 (mg/mL)	Polyvinylpyrrolidone PVP (mg/mL)
4	8.34	5.56	x
9	18.75	12.50	10.75
18	37.50	25.00	21.50
36	75.00	50.00	43.00
54	112.50	75.00	64.50

Compounds for MMC were diluted in DMEM medium (Thermo Fisher Scientific, Gibco, Waltham, MA, USA; Cat. No. 52100-021) with 25 mM HEPES and were filtered through an SFCA 0.45 μm filter (431220, Corning, New York, USA). Then 40 μg/mL of gentamicin (Lek Pharmaceuticals, Ljubljana, Slovenia) and 10% fetal bovine serum (FBS; Thermo Fisher Scientific, Gibco, Waltham, MA, USA; Cat. No. 10270-106) were added in a volume accounted for in the calculation of the final concentration of MMC compounds. l-ascorbic acid-2-phosphate trisodium salt (Asc-2P; 49752, Sigma-Aldrich, USA) at 50 μg/mL was added to each of the MMC media to stimulate collagen synthesis.

### Antifibrotic Drug Treatment

Only the 18% FVO concentration of the Ficoll mix (Fc18) was used in the drug testing experiments. The compound was diluted as described above, with the same supplements, and was sterilized with an SFCA 0.20 μm filter (431219, Corning, New York, USA). Minoxidil (MXD) (M4145, Sigma-Aldrich, USA) was dissolved in 96% ethanol (EtOH) to a stock solution of 119 mM, and was stored at −20 °C in aliquots. The working concentrations of MXD (0, 0.25, 0.5, 0.75, 1, 2 mM) were diluted immediately before each experiment in a complete medium with or without MMC. Fresh media were prepared before each experiment.

### Cell Culture

Primary fibroblast cells from the medial and lateral side of clubfoot (CF-M, CF-L) were expanded in DMEM medium with 25 mM HEPES and 20% fetal bovine serum (FBS) and 40 μg/mL gentamicin in an incubator (37 °C, 8% CO_2_) prior to the start of the experiments. Neonatal normal human dermal fibroblasts (NHDF; CC-2509, Lonza, Basel, Switzerland) were used as a reference cell culture as they have been frequently used in similar studies. NHDFs were cultivated under the same conditions as clubfoot cells in the same culture medium that contained 10% FBS.

The cells for the experiments were seeded in polystyrene cell culture well plates (TPP, Techno Plastic Products, Switzerland) or into glass bottom black cell culture 24 well plates (P24-1.5H-N, Cellvis, Mountain View, CA, USA) suitable for microscopy imaging. Cell seeding density of 10,000 or 20,000 cells/cm^2^ was used, depending on the type of experiment. Seeding density of 10,000 cell/cm^2^ was used when we preferred to evaluate the proliferation of cells in different environments (initial MMC experiments, resazurin assay). Seeding density of 20,000 cells/cm^2^ was used for gene expression, and second-harmonic generation (SHG) microscopy, and experiments were used to measure ECM production. Cell number and viability was determined by the Vi-CELL XR Analyzer (Beckman Coulter, USA). After 24 h, the cell culture medium was replaced with the medium supplemented with 10% FBS, 50 μg/mL Asc-2P, with/without the addition of MMC and with/without MXD. The addition of MXD was considered as the start day (day 0) of treatment for all experiments. The duration of the experiments ranged from 4 to 16 days, and is specified in the respective Methods section. The cell culture media were changed every 3 to 4 days. The experiments with MXD were conducted only with the selected MMC media group (Fc18) and with cells from the contracted medial side (CF-M).

The cell seeding densities and the cultivation conditions for the experiments in the ECIS real-time analysis system differ from all other experiments. We described them in detail in Supporting Information Section 2.

### Proliferation and Cytotoxicity Assays

Detailed experimental procedure of cell mitochondrial activity measurement (resazurin assay), ECIS (real-time, label-free cellular analysis) measurement and PicoGreen dsDNA assay are described in Supporting Information Section 2.

### Immunofluorescence Staining and Microscopy

Extracellular deposition of type I collagen was detected by immunofluorescence staining on day 7 after seeding the cells into 24-well glass bottom black cell culture plates (P24-1.5H-N, Cellvis, Mountain View, CA, USA). Cells were rinsed with phosphate-buffered saline solution (PBS) with 5% FBS and were kept on ice. Then, a primary rabbit polyclonal anticollagen type I antibody (1:1000 in PBS with 5% FBS; LSL-LB-1197, Cosmo Bio, Japan) was added for 2 h incubation on ice, protected from light. Subsequently, the cells were rinsed again with PBS with 5% FBS, were removed from the ice, and were fixed with 2% paraformaldehyde in PBS solution with 5% FBS for 20 min. The cells were again rinsed with PBS and were incubated in PBS with 1% FBS for another 20 min to ensure blocking of nonspecific binding sites. After washing with PBS, a secondary antibody Alexa Fluor 488-conjugated F(ab′)2-Goat anti-Rabbit IgG (1:400 in PBS with 1% FBS; A-11070, Invitrogen, Thermo Fisher Scientific, USA) was added for 1 h at room temperature together with Hoechst #33258 (5 μg/mL in PBS; 94403, Sigma-Aldrich, USA), which penetrates through the nonpermeabilised cell membrane to stain the cell nuclei. The fluorescence signal was visualized by an Olympus IX51 epifluorescence microscope with a DP74 camera (both Olympus Corp., Japan). The image acquisition settings were kept identical for all wells.

### Analysis of Fluorescence Images

Microphotographs obtained from the microscope (at least 10 per well, in triplicate) were analyzed using ImageJ FIJI software^54^ (v.1.54f). Differences in the fluorescence signal of type I collagen were measured after automatic background subtraction as an integrated fluorescence density in the entire image area. The amount of deposited type I collagen was expressed as the integrated fluorescence density value (IntDen), normalized to the mean value measured in the Ctrl wells (nontreated cells cultivated in media without MMC). The number of cells in each image was determined from the staining of the nuclei, which were counted automatically using the ImageJ FIJI StarDist plugin.^55^

### Second-Harmonic Generation Imaging

Cells for second-harmonic generation (SHG) imaging were seeded in 24-well glass bottom black cell culture plates (P24-1.5H-N, Cellvis, Mountain View, CA, USA) and were analyzed on day 7 of cultivation in MMC media. The cells were kept alive for the imaging, as this method is label-free, and fixation would reduce the SHG signal. The SHG signal was obtained on a confocal microscope in order to determine the presence of properly structured type I collagen fibers. Cells in the glass bottom well plate were visualized using a 63× water immersion objective (HC PL APO CS 2 63x/1.20 Water), mounted on a Leica DMi8 inverted microscope with a Leica TCS SP8 X confocal unit (Leica Microsystems, Germany). A Chameleon Discovery TPC pulsed femtosecond laser (Coherent Inc., USA; 860 nm tunable output, 80 MHz, 1.6 W) was used to generate SHG (green pseudocolour) and autofluorescence (red pseudocolour) signals. Leica nondescanned HyD detectors and a 430/24 band-pass filter were used for collecting the SHG signals, and Leica nondescanned HyD detectors and a 610/75 band-pass filter were used for collecting the autofluorescence signals.

### Sircol Assay

To analyze the pattern of collagen deposition in cell culture under MMC (Fc18) conditions, different sample fractions were extracted (media fraction, pepsin/acid-soluble ECM fraction, and cross-linked insoluble ECM fraction). The cells were grown in 6-well polystyrene plates for 16 days to ensure a sufficient amount of cell-deposited ECM for the analysis. Low protein binding collection tubes (90410, 1.5 mL, Thermo Scientific, USA) were used for handling samples throughout the experiment to minimize loss of the material. The Sircol Soluble Collagen Assay Kit (S1000, BioVendor, UK) was used to analyze the collagen content in the media fraction and in the pepsin/acid-soluble ECM fraction of the samples.

The fraction of soluble collagen deposited in the media was measured in the media conditioned by the cells cultured with/without MMC (Fc18) for at least 4 days after the last media change until the end of the experiment. One mL of conditioned media per sample was used for analysis, together with a fresh sample of the corresponding media type as a blank. The analysis procedure was the same as for samples from the pepsin/acid-soluble collagen fraction (described below), with the omission of the pepsin digestion step. Fresh media from each group were used as blanks to account for any interference of noncollagenous proteins (e.g., from serum).^56^

The fraction of pepsin/acid-soluble collagen in ECM was extracted and analyzed as follows: Cell layers were rinsed with PBS. Collagen was recovered from the cell layers by acidic pepsin digestion (0.1 mg/mL in 0.5 M acetic acid; pepsin EC 3.4.23.1. from Sigma-Aldrich, USA) in a cold room, on a shaker. After overnight incubation, the cell layers were harvested with a cell scraper, transferred to low protein binding tubes and centrifuged. The pepsin insoluble pellet was stored at 4 °C for later use, while the supernatant was used to continue the soluble Sircol assay according to the kit manufacturer protocol. The amount of collagen in the samples was then determined from the standard curve based on the binding of Sirius Red dye and was normalized to the untreated control samples (Ctrl).

The pellets of the insoluble collagen fraction from each sample were analyzed with the Hydroxyproline assay, as well as a parallel set of samples for the detection of total collagen content.

### Hydroxyproline Assay

Cells were washed with PBS, lysed in RIPA buffer (R0278, Sigma-Aldrich, USA), harvested with a cell scraper and collected into heat-resistant polypropylene screw cap tubes. After sonication, an aliquot of each sample was used to perform a Pierce BCA assay (23225, ThermoFisher Scientific, USA) according to the manufacturer’s instructions to measure the total protein content in the sample.

The rest of each sample was then digested in 6 N HCl at 120 °C for 3 h. The amount of collagen deposited in the extracellular matrix was determined as the hydroxyproline content in the sample, using the Hydroxyproline Colorimetric Assay Kit (MAK008, Sigma-Aldrich, USA) according to the manufacturer’s instructions. Both assays were measured using a VersaMax Absorbance Microplate Reader (Molecular Devices, USA) at 560 nm. The amount of hydroxyproline is customarily used to calculate the total collagen content in cell culture or tissue samples, which can be extrapolated by multiplying the sample hydroxyproline by a factor of 6.6.^57^

In the experiments with MXD, the cells were grown at the same density, but in 12-well plates. A whole sample was analyzed, representing both intracellular collagen and collagen deposited in the extracellular matrix layer. Thus, the relative configuration of hydroxyproline is normalized to the total protein content per sample. In experiments to determine the total amount of insoluble collagen in the corresponding sample fraction, the total protein content could not be measured. The data are therefore presented as the relative concentration of hydroxyproline.

### Enzyme-Linked Immunosorbent Assay (ELISA)

The concentration of pyridinoline and deoxypyridinoline cross-links in the cell-produced extracellular matrix of samples with/without MXD treatment was measured by ELISA, using the MicroVue PYD Enzyme Immunoassay kit (8010, MicroVue Quidel, San Diego, CA, USA) according to the manufacturer’s instructions. Aliquots of samples previously analyzed by the hydroxyproline assay were used for these measurements. The concentration of the cross-links was normalized to the concentration of hydroxyproline and total protein in the same sample.

### RNA Isolation and Real-Time PCR

Real-time PCR was performed to assess differences in the relative expression of mRNA of selected genes. After 24 h of cultivation with/without MMC (Fc18) and MXD treatment, the cell RNA was isolated using the Total RNA Purification Micro Kit Plus (48500, Norgen Biotek, Canada). A NanoDrop One^C^ microvolume UV–vis spectrophotometer (Thermo Scientific, USA) was used to check RNA quality and quantity. RNA at a concentration of 300 ng/μL was used for reverse transcription into cDNA by the Omniscript Reverse Transcription Kit (205113, Qiagen, Germany) with a Random Primer Mix (S1330S, New England BioLabs, USA). The reaction was performed in a T-Personal Thermocycler (Biometra, Germany).

Real-time PCR was performed using 5xHOT FIREPol Probe qPCR Mix Plus (ROX) (08-14-00008, Solis BioDyne, Estonia) and TaqMan Gene Expression Assays (Life Technologies, USA) labeled with the FAM reporter dye specific to target the following human genes: the extracellular matrix proteins collagen type I α-1 chain (*COL1A1*; Hs00164004_m1), collagen type I α-2 chain (*COL1A2*; Hs00164099_m1), collagen type III α-1 chain (*COL3A1*; Hs00943809_m1), collagen type VI α-3 chain (*COL6A3*; Hs0091523_m1), and fibronectin 1 (*FN1*; Hs01549976_m1), the enzymes catalyzing collagen post-translational modifications lysyl hydroxylase 1 (*PLOD1*; Hs00386343_m1), lysyl hydroxylase 2 (*PLOD2*; Hs01118184_m1), lysyl hydroxylase 3 (*PLOD3*; Hs01126612_m1), lysyl oxidase (*LOX*; Hs00942483_m1), and prolyl 4-hydroxylase (*P4HTM*; Hs00977922_m1), the pro-fibrotic cytokine transforming growth factor β-1 (*TGFB1*; Hs00998133_m1), the cytoskeletal protein α-smooth muscle actin (*ACTA2*; Hs00909449_m1), the collagen degradation enzymes matrix metalloproteinase 2 and 9 (*MMP2, MMP9*; Hs01548727_m1, Hs00957562_m1), and a reference gene β-actin (*ACTB*; Hs99999903_m1).

The final reaction was conducted using the LightCycler 480 Instrument (Roche, Switzerland). The total reaction volume was 20 μL, and the cycle parameters were as follows: 2 min at 50 °C, 10 min at 95 °C, 40 cycles of 15 s at 95 °C and 1 min at 60 °C. The results are presented as 2^∧^(−ΔΔCt). Data were normalized against a reference gene and according to the gene expression in the untreated control sample (Ctrl).

### Immunohistochemical Staining

The data presented here complement our previously published data in the study by Novotny et al.,^19^ and are used together to discuss points raised in this study. The immunohistochemistry staining was performed on the same set of patients’ tissues as in the study mentioned above and with the same methods. Briefly, tissue samples from the medial side (M-side) and the lateral side (L-side) of 10 patients with relapsed clubfoot were fixed and embedded in paraffin. Primary antibodies anticollagen III (C7805, Sigma; 1:500, 1 h at room temperature), anticollagen VI (ab6588, Abcam; 1:200, 30 min at room temperature), anti-LH1 (ab262947, Abcam; 1:100, 1 h at room temperature), anti-Lhx2/LH2 [CL6137] (ab243030, Abcam; 1:500, 1 h at room temperature), anti-PLOD3 (LH3) (ab128698, Abcam; 1:400, overnight at 4 °C) were used for detection by immunohistochemistry (IHC). Antigen retrieval, hydrogen peroxide block, protein block, secondary antibody reaction, and visualization were performed according to the Abcam protocol by applying the EXPOSE Mouse and Rabbit Specific HRP/DAB IHC Detection Kit (ab236466, Abcam, Cambridge, UK). The slides were counterstained with hematoxylin. The positivity of the detected antibodies was evaluated using a light microscope, and was subsequently quantified with image analyzer signal thresholding in the NIS Elements 3.0 AR program (Laboratory Imaging, Czech Republic). The number of pixels within the signal range was quantified from 10 independent areas of each sample and was used to calculate the mean percentage of the positive area in each sample. Mean percentage values for each patient are presented in scatter plots and were statistically compared across the experimental groups with a paired *t* test.

### Statistical Analysis

SigmaStat 4.0 (Systat Software Inc., USA) or GraphPad Prism 10 (GraphPad Software, USA) was used to check normality and variance (homoscedasticity) of the data and to perform statistical analyses. A parametric statistical test with an appropriate posthoc test (i.e., Paired *t* test, One-Way ANOVA with Dunnet’s or Tukey’s test for multiple comparisons, Unpaired *t* test with Welch’s correction) were used if conditions for normality and equal variance of data were met. If these assumptions were violated, a nonparametric test was used (i.e., a Kruskal–Wallis test with Dunn’s multiple comparisons test). Two-Way ANOVA with Tukey’s test for multiple comparisons was used to analyze the data from experiments with/without an MMC environment and minoxidil, when applicable. Significance of the differences was set at *p* < 0.05 unless stated otherwise. Formats in which the data are presented are specified in the respective figure captions along with the statistical tests that were used. All graphs were created in GraphPad Prism.

## Results

### MMC Controls Cell Proliferation and Metabolic Activity Depending on MMC Concentration and Cell Type Sensitivity

**Figure 1:**
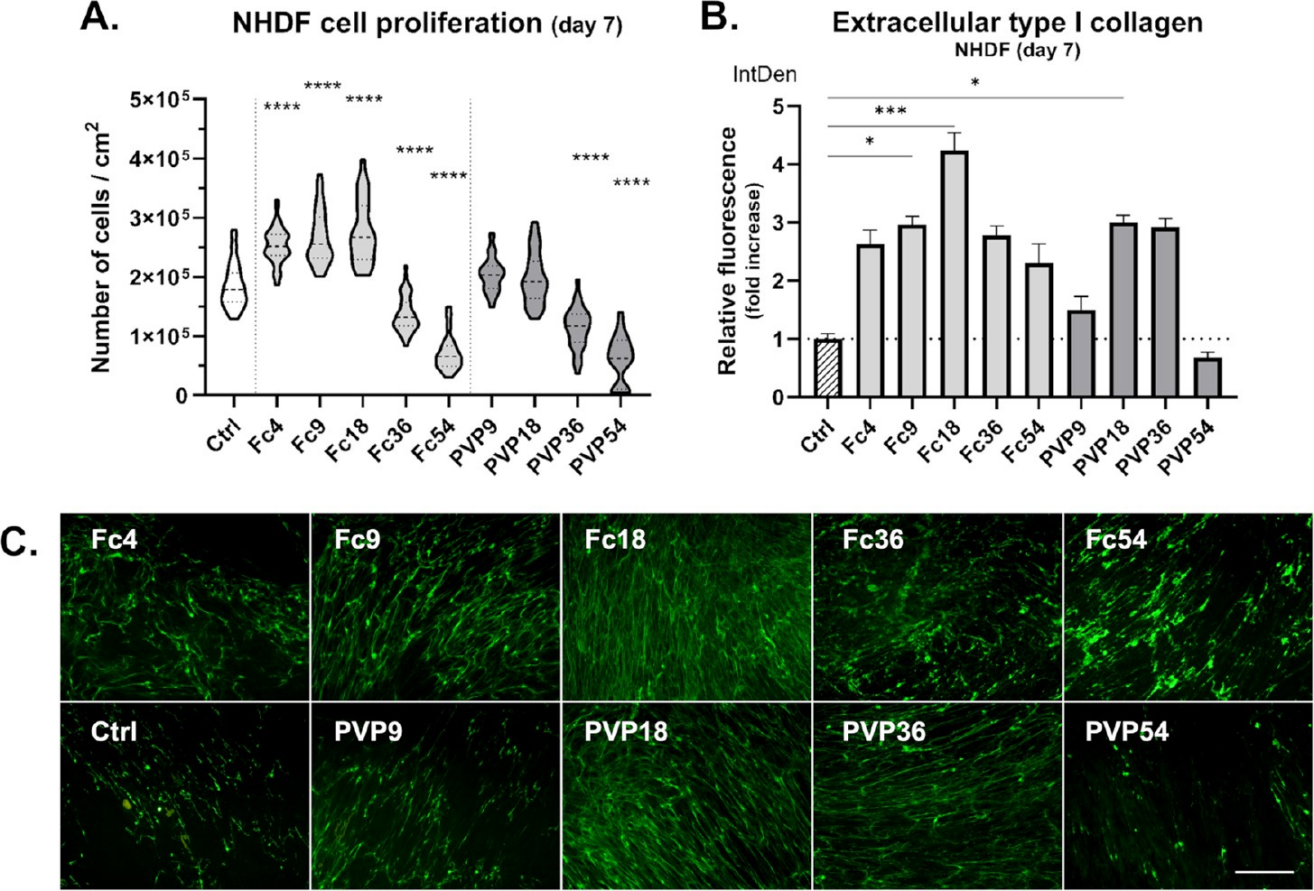
Proliferation and extracellular type I collagen deposition of **NHDF** fibroblasts after 7 days of culture with/without MMC. (A) Cell proliferation in media with different MMC concentrations. Truncated violin plot, median with IQR (*n* = 18–137). Kruskal–Wallis ANOVA, Dunn’s test (vs Ctrl). (B) Integrated fluorescence density of extracellular collagen relative to Ctrl (no MMC). Mean + SEM (*n* = 5–6). Kruskal–Wallis ANOVA, Dunn’s test (vs Ctrl). (C) Demonstrative images of extracellular type I collagen deposition after fluorescent staining. Olympus IX51 microscope, 10× objective. Scale bar = 200 μm.

**Figure 2:**
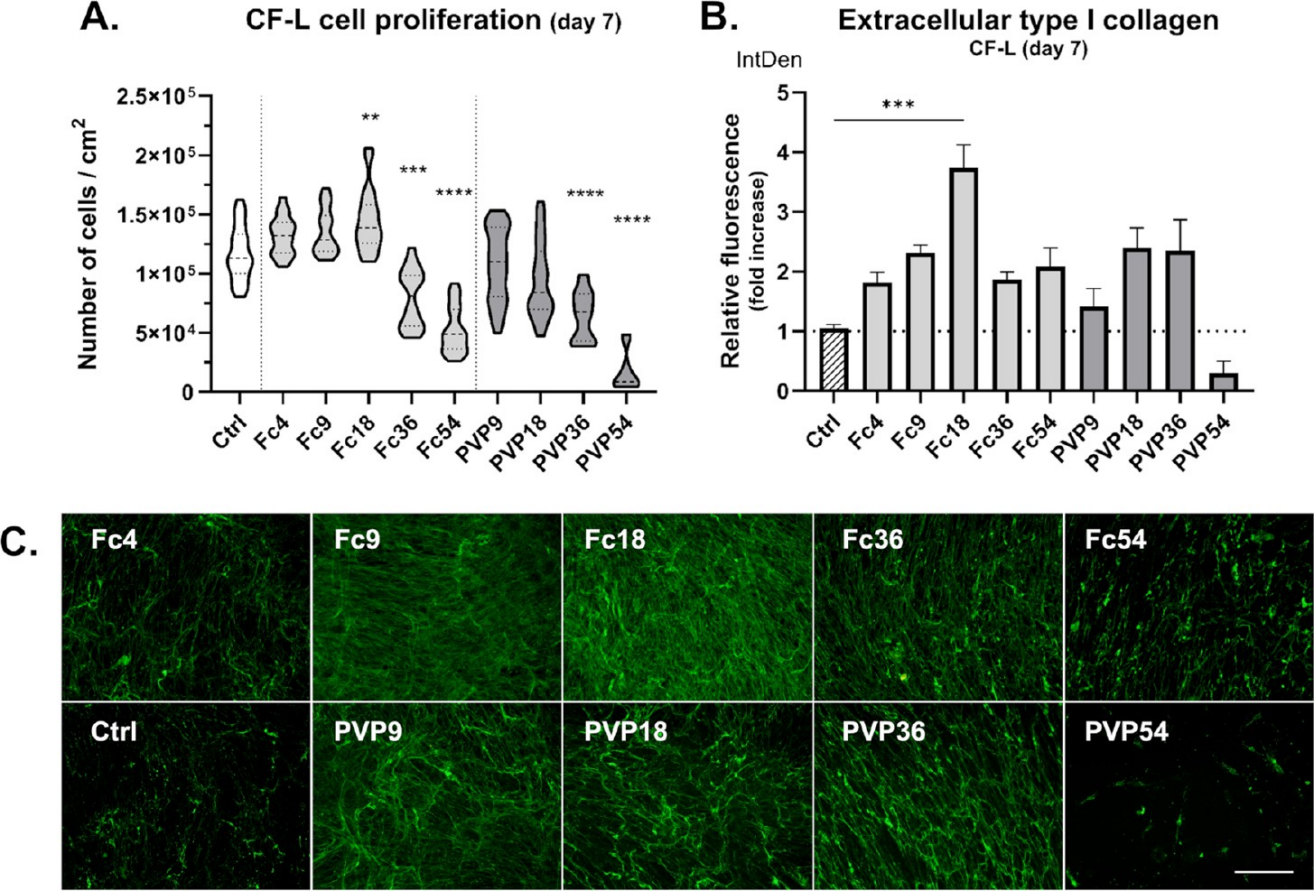
Proliferation and extracellular type I collagen deposition of noncontracted **clubfoot-derived cells (CF-L)** after 7 days of culture with/without MMC. (A) Cell proliferation in media with different MMC concentrations. Truncated violin plot, Median with IQR (*n* = 10–54). Kruskal–Wallis ANOVA, Dunn’s test (vs Ctrl). (B) Integrated fluorescence density of extracellular collagen relative to Ctrl (without MMC). Mean + SEM (*n* = 5–6). Kruskal–Wallis ANOVA, Dunn’s test (vs Ctrl). (C) Demonstrative images of extracellular type I collagen deposition after fluorescent staining. Olympus IX51 microscope, 10× objective. Scale bar = 200 μm.

**Figure 3:**
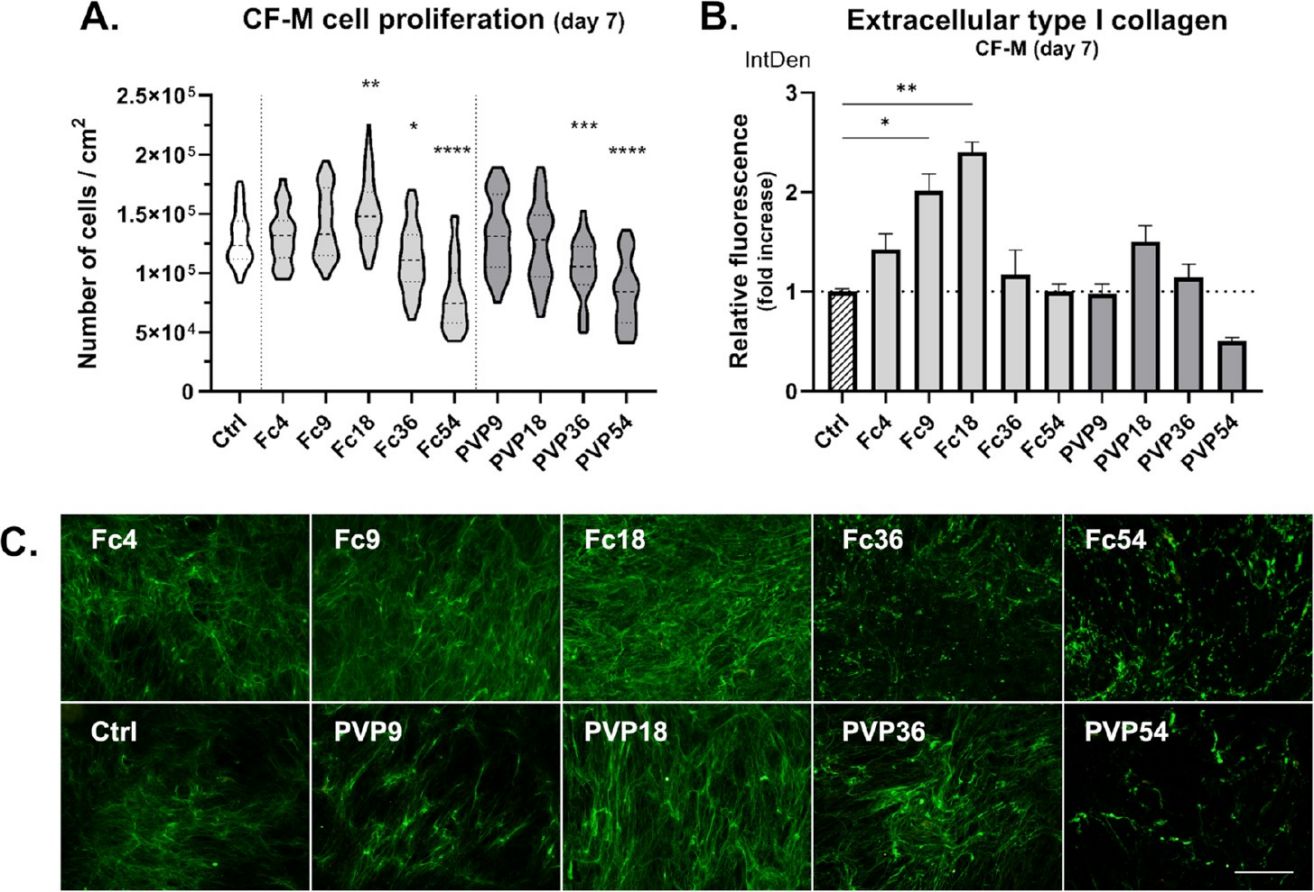
Proliferation and extracellular type I collagen deposition of contracted **clubfoot-derived cells (CF-M)** after 7 days of culture with/without MMC. (A) Cell proliferation in media with different MMC concentrations. Truncated violin plot, median with IQR (*n* = 24–75). Kruskal–Wallis ANOVA, Dunn’s test (vs Ctrl). (B) Integrated fluorescence density of extracellular collagen relative to Ctrl (without MMC). Mean + SEM (*n* = 5–7). Kruskal–Wallis ANOVA, Dunn’s test (vs Ctrl). (C) Demonstrative images of extracellular type I collagen deposition after fluorescent staining. Olympus IX51 microscope, 10× objective. Scale bar = 200 μm.

First, we investigated the effect of Ficoll 70 kDa + Ficoll 400 kDa mix (Fc) and Polyvinylpyrrolidone 40 kDa (PVP) in a range of concentrations (Fc 4–54, PVP 9–54% FVO v/v in the media) on all three cell types. We seeded primary cell cultures of clubfoot-derived fibroblasts from the fibrotic contracted tissue of relapsed patients (CF-M) and from noncontracted clubfoot tissue from the opposite side of the foot (CF-L) in parallel with normal human dermal fibroblasts (NHDFs). We observed the native state of the cells on day 4 and their metabolic activity (viability) on day 4, 7, and 11. The detailed analysis and results are described in Supporting Information Section 3.1, Figure S1. We quantified the number of cell nuclei in samples on day 7 (Figures 1A, 2A, 3A).

All three cell types proliferated well in both tested MMC agents up to 18% FVO. However, concentrations of 36% FVO (Fc36, PVP36) and especially 54% FVO (Fc54, PVP54) appeared to inhibit the proliferation of all cell types. This inhibition was stronger for NHDFs and CF-L cells, where both the proliferation and the viability of cells grown in PVP36 and PVP54 were relatively low. The cell density on day 7 was in accordance with the metabolic activity of CF-M cells in these MMC concentrations (Figure S1B). Interestingly, on day 7, the Fc at 4–18% FVO significantly enhanced the proliferation of NHDF, but only Fc at 18% significantly increased the proliferation in CF-L and CF-M cells (Figures 1A, 2A, 3A).

### MMC Enhanced Collagen Deposition and Collagen Supramolecular Assembly in the Cultures of Clubfoot-Derived and NHDF Fibroblasts

We compared collagen production by cells grown under MMC conditions by fluorescent staining of type I collagen deposited in the extracellular matrix after 6 days of treatment (7 days in total). We observed slight to significant increases of collagen in both MMC media types (Fc, PVP) and concentrations across all cell types with the exception of PVP54, in which we could see a minimal amount of extracellular collagen (Figures 1, 2, 3B,C). Most likely, this could be attributed to the lowest number of viable cells growing in PVP54 wells. The collagen deposited by cells in PVP54 appeared to be in a more granulated form rather than in a fibrillar form, which also often seemed to be the case with Fc54, and to some extent with Fc36. In general, our observation can be correlated with the relatively low density of cells in particular optical fields. In contrast, we detected the highest extracellular collagen production in Fc18 in all cell types, with Fc9 as the second highest in two of them (Figures 1, 2, 3B,C). We compared the relative integrated fluorescence density (IntDen) of extracellular collagen from fluorescence microphotographs of the cells cultivated with MMC and the control cells in a standard medium (Ctrl = 1). NHDF cells in Fc18 had the highest relative collagen production (value of 4.24). It was lower in CF-L (Fc18) (value of 3.73) and lowest in CF-M (Fc18) (value of 2.40). We also detected a significant increase in collagen production in Fc9, where NHDF (Fc9) reached a value of 2.96 and CF-M (Fc9) reached a value of 2.01 relative to Ctrl. NHDFs was the only cell type in which we detected a significant increase in collagen deposition in PVP18, which was 3.00 relative to Ctrl (Figures 1, 2, 3B). Although the choice of the best macromolecular crowder to promote ECM deposition appears to be cell-specific to some extent, our findings are consistent with the frequent use of 18% FVO crowding concentration in other studies with fibroblasts.^31,33,38,42,43^

**Figure 4:**
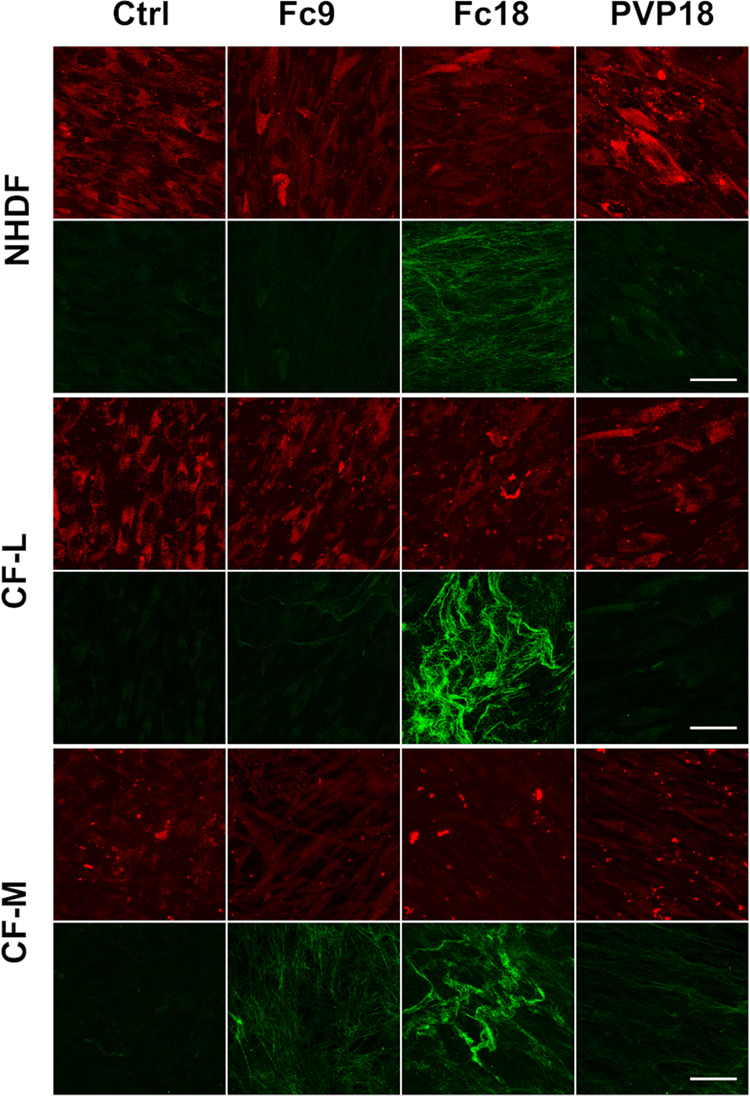
Maturation of collagen fibers. SHG imaging of type I collagen fibers after 7 days of cultivation of normal human fibroblasts (NHDFs) and clubfoot-derived fibroblasts (CF-L, CF-M) with (Fc9, Fc18, PVP18) or without (Ctrl) macromolecularly crowded media - demonstrative images. The red signal (upper row) is the autofluorescence of cells. The green signal (lower row) is SHG. Leica DMi8 inverted microscope with a Leica TCS SP8 X confocal unit, 63× water immersion objective. Scale bar = 50 μm.

To further examine collagen deposition, we imaged live cells grown in the same experimental setup with label-free confocal microscopy visualizing the SHG signal of collagen fibers. SHG offers qualitative information about collagen supramolecular assembly, which was indeed reported to be accelerated in MMC conditions.^58^ The SHG signal revealed that in just a 7 day experiment the Fc18 media accelerated structural maturation and the assembly of collagen fibers in all cell types to a detectable level (Figure 4). According to our experience, a strong SHG signal can usually be detected in a fibroblast cell culture after 3 weeks of cultivation,^22,59^ a time which was significantly shortened to a week with the use of MMC in a cell culture.

The three tested cell types (NHDFs healthy fibroblast control, clubfoot noncontracted control CF-L, and CF-M from fibrotic clubfoot tissue) exhibited similar behavior under MMC conditions. We therefore continued the experiments only with the Fc18 media, which was the best-performing MMC combination, and with CF-M cells to explore the advantageous effect of the MMC microenvironment in the development of an *in vitro* model of clubfoot fibrosis.

### MMC (Fc18) Supported Higher Conversion of Soluble Collagen into Insoluble Collagen in the Culture of Clubfoot-Derived Fibroblasts

**Figure 5:**
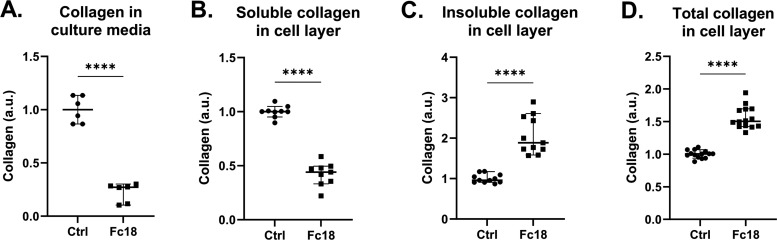
Analysis of collagen deposition by clubfoot-derived fibroblasts (CF-M) in distinct fractions of the sample during 16-day cultivation without (Ctrl) or with (Fc18) macromolecularly crowded media. (A) Collagen produced into cultivation media between media changes (*n* = 6). (B) Soluble collagen newly deposited into the extracellular matrix of the cell layer (*n* = 9). (C) Insoluble cross-linked collagen deposited into the extracellular matrix (*n* = 11). (D) Total collagen deposited in the cell layer extracellular matrix (*n* = 14). Scatter plots, Median with 95% CI. Paired *t* test (****p < 0.0001).

Some limitations to the use of primary patient’s cells in clubfoot research are the relative scarcity of samples and limited access to large amounts of cells of low passages. The higher mean population doubling time of CF-M (approximately 30 h^22^) in comparison to NHDFs (approximately 24 h) and CF-L (approximately 27 h, data not shown) in standard cultivation conditions also adds to the prospect of longer cultivation times to generate enough extracellular matrix for specific analyses. To quantify the stages of collagen production and incorporation into the extracellular matrix, we cultivated CF-M with and without Fc18 for 16 days. Subsequently, we measured the amount of collagen in distinct fractions of a sample using Sircol and Hydroxyproline assays. The microenvironment created by modifying the culture medium with MMC promoted the conversion of soluble collagen toward nonsoluble collagen and accelerated its deposition into the extracellular matrix. The amount of soluble collagen measured in the culture medium was 3.7 times lower in Fc18 than in Ctrl (Figure 5A). Collagen newly deposited into the extracellular matrix and recently cross-linked collagen (soluble in pepsin and acetic acid solution) was 2.3 times lower in the Fc18 group (Figure 5B). In contrast, the amount of cross-linked, insoluble collagen deposited into the cellular layer was 2.0-fold higher in Fc18 (Figure 5C). The total amount of collagen deposited into the cell layer after 16 days of cultivation was 1.5-fold higher in the Fc18 group than in Ctrl (Figure 5D).

### MMC (Fc18) Stimulated the Expression of Fibrosis-Related Genes in Clubfoot-Derived Fibroblasts

**Figure 6:**
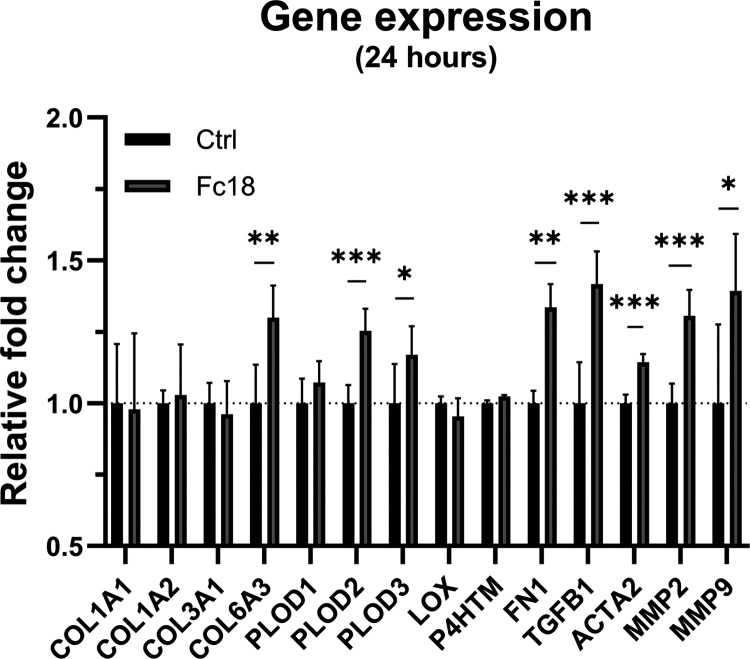
Relative mRNA expression of genes in clubfoot-derived fibroblasts (CF-M) after 24 h of cultivation without (Ctrl) or with (Fc18) a macromolecularly crowded environment. Genes of interest: *COL1A1*, *COL1A2* (collagen type I α-1 and α-2 chains); *COL3A1* (collagen type III α-1 chain); *COL6A3* (collagen type VI α-3 chain); *PLOD1*, *PLOD2*, *PLOD3* (lysyl hydroxylase 1, 2, and 3); *LOX* (lysyl oxidase); P4THM (prolyl-4-hydroxylase); *FN1* (fibronectin 1); *TGFB1* (transforming growth factor-β 1); ACTA2 (α-smooth muscle actin); *MMP2*, *MMP9* (matrix metalloproteinases 2 and 9). Normalized against *ACTB* (β-actin) reference gene. Data presented as 2^∧^(−ddCt), relative to Ctrl (calibrator). Mean + SD (*n* = 3–5). Unpaired *t* test with Welch’s correction (*p < 0.05, **p < 0.01, ***p < 0.001).

At the mRNA level, clubfoot cells reacted to the addition of Fc18 media as early as 4 h after the media change with a slight but insignificant increase in gene expression (data not shown). However, after 24 h (Figure 6), the levels had risen significantly for genes *PLOD2*, *PLOD3* (encoding lysyl hydroxylase 2 and 3—the enzymes catalyzing collagen post-translational modifications and cross-linking), *FN1* (fibronectin 1, i.e., an extracellular matrix protein), *TGFB1* (transforming growth factor β-1, i.e., a pro-fibrotic cytokine), *ACTA2* (α-smooth muscle actin, i.e., a cytoskeletal protein, marker of myofibroblast formation), and also *COL6A3* (collagen type VI α 3 chain), *MMP2* and *MMP9* (matrix metalloproteinases 2 and 9—collagen degradation enzymes). By contrast, the expression of genes for other tested extracellular matrix proteins such as procollagen structural components *COL1A1*, *COL1A2* (collagen type I α-1 and α-2 chains), *COL3A1* (collagen type III α-1 chain), and other collagen modifying enzymes *PLOD1* (lysyl hydroxylase 1), *LOX* (lysyl oxidase) and *P4THM* (prolyl 4-hydroxylase) remained unchanged or exhibited large variance both in Ctrl and in the Fc18 medium. No significant decrease in gene expression of the measured markers was detected in Fc18. These results indicate that MMC, specifically in Fc18 form, shifts CF-M fibroblasts toward pro-fibrotic phenotype without any other supplementation.

**Figure 7:**
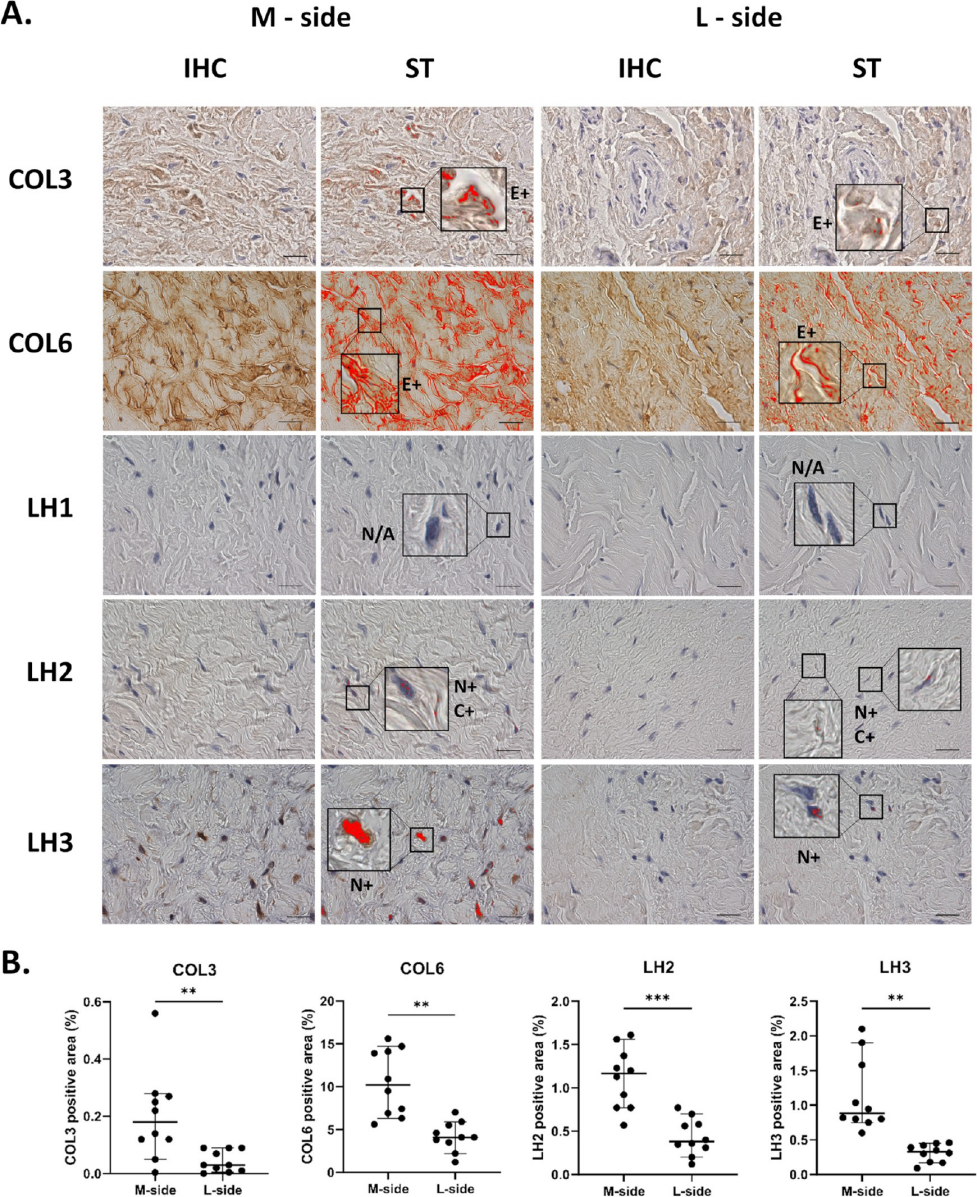
Comparison of contracted (M-side) and noncontracted (L-side) of clubfoot tissue—complementary data. (A) Demonstrative images of immunohistochemical (IHC) staining and signal detection. Staining of collagen type III and VI, lysyl hydroxylases 1, 2, and 3 (LH1, LH2, LH3). The red areas in the ST (signal threshold) columns represent the positive pixels after thresholding. An image analyzer was then used to measure the percentage of the area with a positive signal from the total area. *E+ Extracellular positivity, N+ Nuclear positivity, C+ Cytosolic positivity*, N/A *Not Assessed.* Scale bar = 20 μm. (B) The percentage of COL3, COL6, LH2, and LH3 positive area after IHC signal quantification. LH1 was not detectable. Scatter plots, median with 95% CI (*n* = 10). Paired *t* test (**p < 0.01, ***p < 0.001).

Interestingly, these results correlated well with previously published analyses of clubfoot tissues in our earlier studies.^17,19^ We performed new complementary immunohistochemistry (IHC) analyses of additional markers on the paraffin-embedded tissue sections previously collected for the study by Novotny et al.^19^ to provide more comprehensive information for comparison with our *in vitro* model. IHC staining against collagen type III, collagen type VI, and collagen-cross-linking enzymes lysyl hydroxylase 2 and 3 (LH2, LH3) showed a significantly increased area of tissue sections positively stained for these antigens in samples of contracted medial side clubfoot tissue when compared to the lateral side tissue (Figure 7). Significant extracellular IHC positivity was detected for both collagen types III and VI, whereas nuclear and cytosolic positivity was detected for LH2, and only nuclear positivity in the case of LH3. Lysyl hydroxylase 1 (LH1) was not detected in the tissues from either side. Related IHC data regarding upregulation in TGF-β 1, α-smooth muscle actin, fibronectin, LOX, MMP2, MMP9 in clubfoot medial tissue were published in Novotny et al.,^19^ and data for type I collagen were published in Knitlova et al.^18^ We therefore refer to them in more detail in the discussion.

### MMC (Fc18) Positively Influenced the Proliferation of Clubfoot-Derived Fibroblasts in the Presence of Minoxidil

In our earlier experiments, we have already determined the MXD concentrations suitable for standard cell culture of clubfoot-derived cells.^22^ Here, we aimed to explore if the dose-dependent inhibitory effect of minoxidil on cell proliferation that we had previously observed follows the same trend when administered in Fc18 media. We generally observed a moderately positive effect on cell proliferation in MMC medium Fc18. We therefore tested a full range of MXD concentrations again, including the highest concentrations, with CF-M cells seeded at a density of 10,000 per cm^2^.

We monitored the effect of the absence or presence of MXD in concentrations of 0, 0.25, 0.50, 0.75, 1, and 2 mM on clubfoot-derived cells in Ctrl and Fc18 media in real-time for 160 h using the Electric Cell–substrate Impedance Sensing device (ECIS) (Figure S2A). Subsequently, we estimated the amount of cellular dsDNA in the samples (Figure S2B). The results are described in detail in the Supporting Information Section 3.2. We confirmed that generally larger amounts of cells are growing in the Fc18 media, i.e., about 30% more than in the Ctrl media in all MXD groups, except for the highest concentration. Based on these analyses, we excluded 1 and 2 mM MXD from further analysis, due to their strong inhibitory effect on cell proliferation.

### The Reduction of Collagen Production, Deposition and Cross-Linking by Minoxidil Treatment was Less Pronounced in MMC (Fc18)

**Figure 8:**
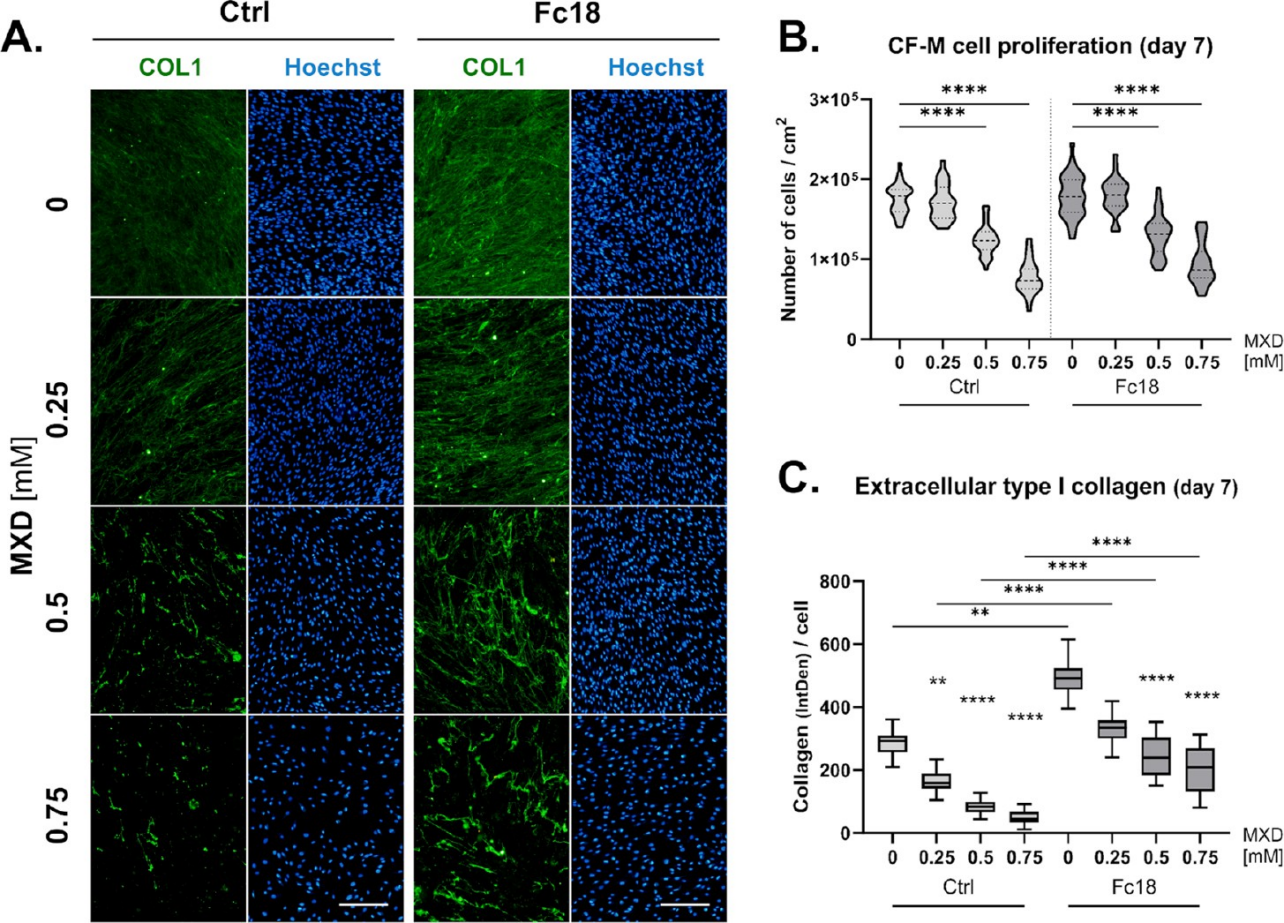
Proliferation and extracellular collagen type I deposition of contracted clubfoot-derived cells (CF-M) after 7 days of culture without (Ctrl) or with (Fc18) macromolecularly crowded media and different concentrations of minoxidil (MXD). (A) Demonstrative images of extracellular type I collagen deposition (green) and cell density (blue - nuclei) after fluorescent staining. Olympus IX51 microscope, 10× objective. Scale bar = 200 μm. (B) Cell proliferation in Ctrl and Fc18 media with different MXD concentrations. Truncated violin plot, Median with IQR (*n* = 30–36). ANOVA, Tukey’s test. (C) The amount of extracellular collagen deposited presented as the integrated fluorescence density of extracellular type I collagen normalized to the cell count. Box plot, Median with IQR (*n* = 30–33). Kruskal–Wallis ANOVA, Dunn’s test (**p < 0.01, ***p < 0.001, ****p < 0.0001).

With a focus on ECM deposition, we performed the rest of the experiments with CF-M cells seeded at a density of 20,000 per cm^2^. Higher initial cell density practically erased the difference between the cell proliferation rates in the Ctrl and Fc18 media on day 7, when no MXD or a low concentration of 0.25 mM MXD was added (Figure 8). We observed a notable difference in a group with 0.75 mM MXD, in which case the cells in Fc18 media proliferated faster by approximately 10% (Figure 8). At these conditions, the deposition of extracellular type I collagen per cell under MXD treatment in the Ctrl media followed the same dose-dependent decrease as previously reported.^22^ An analysis of microphotographs from fluorescence microscopy showed that the Fc18 medium increased the total amount of collagen deposited in all groups. However, the proportional differences between individual MXD concentrations and the control without MXD are lower in the Fc18 medium compared to the same concentrations in the Ctrl medium. The amount of deposited extracellular collagen in the Ctrl group was reduced to 54, 28, and 15% by the addition of 0.25, 0.5, and 0.75 mM MXD, respectively. In the Fc18 group, the respective reductions were to 68, 49, and 43% relative to the nontreated samples (Figure 8C). A comparison between the collagen production after 7 days in Fc18 and the production measured in a standard cell culture after 21 days in Knitlova *et* Doubkova et al.^22^ suggests that the environment created by the Fc18 media enhanced and speeded up collagen deposition. The proportional amount of collagen that was deposited in Fc18 with higher MXD concentrations is closer to values observed at a later time intervals in standard cell culture.^22^ Both of the higher concentrations of MXD (0.5 and 0.75 mM) significantly reduced the collagen content.

**Figure 9:**
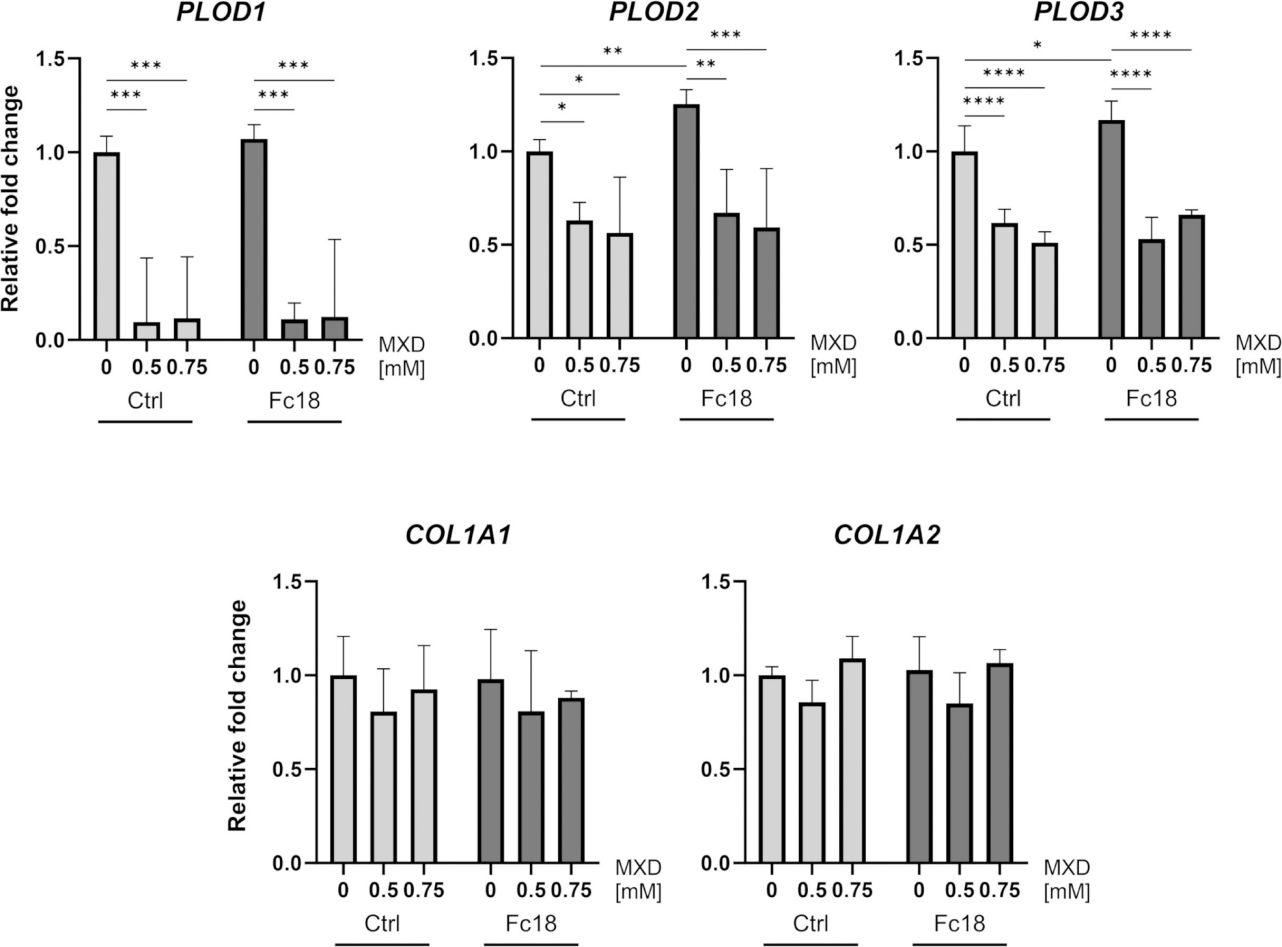
Relative mRNA expression of genes for collagen and collagen-cross-linking enzymes in clubfoot-derived fibroblasts (CF-M) after 24 h of cultivation in noncrowded Ctrl or macromolecularly crowded Fc18 media with or without the addition of minoxidil (MXD). Genes of interest: *PLOD1*, *PLOD2*, *PLOD3* (lysyl hydroxylase 1, 2, and 3); *COL1A1*, *COL1A2* (collagen type I α-1 and α-2 chains). Normalized against *ACTB* (β-actin) reference gene. Data presented as 2^∧^(−ddCt), relative to Ctrl (calibrator). Mean + SD (*n* = 4). Two-Way ANOVA, Tukey’s multiple comparisons test (*p < 0.05, **p < 0.01, ***p < 0.001, ****p < 0.0001).

We measured the expression level of related genes to examine the effect of Fc18 media with/without MXD on type I procollagen structural components and enzymes active in collagen post-translational modifications. After 24 h of treatment in both Fc18 and in Ctrl, the expression of genes for lysyl hydroxylase isoforms 1, 2, and 3 (LH1, LH2, LH3; encoded by genes *PLOD1*, *PLOD2*, *PLOD3*) were downregulated significantly and to a similar extend by 0.5 and 0.75 mM MXD (Figure 9). The downregulation was slightly more pronounced in the *PLOD1* and *PLOD3* expression in the Fc18 group, but we observed a more significant decrease in *PLOD2* expression levels after MXD treatment in Fc18 than in the Ctrl media. It was previously reported that the effect of minoxidil on specific lysyl hydroxylase isoforms varies.^60^ In clubfoot-derived cells, the relative expression of *PLOD1* was reduced the most, followed by *PLOD3* and *PLOD2*. The genes for procollagen α chains (*COL1A1*, *COL1A2*) were not significantly affected by the addition of MXD or by the type of medium. The inhibitory effect of minoxidil on collagen is thus confirmed to be largely dependent on the disruption of collagen fibrillogenesis in terms of downregulating the collagen cross-linking enzyme expression, not via reduction of its precursors in the form of procollagen chains.

**Figure 10:**
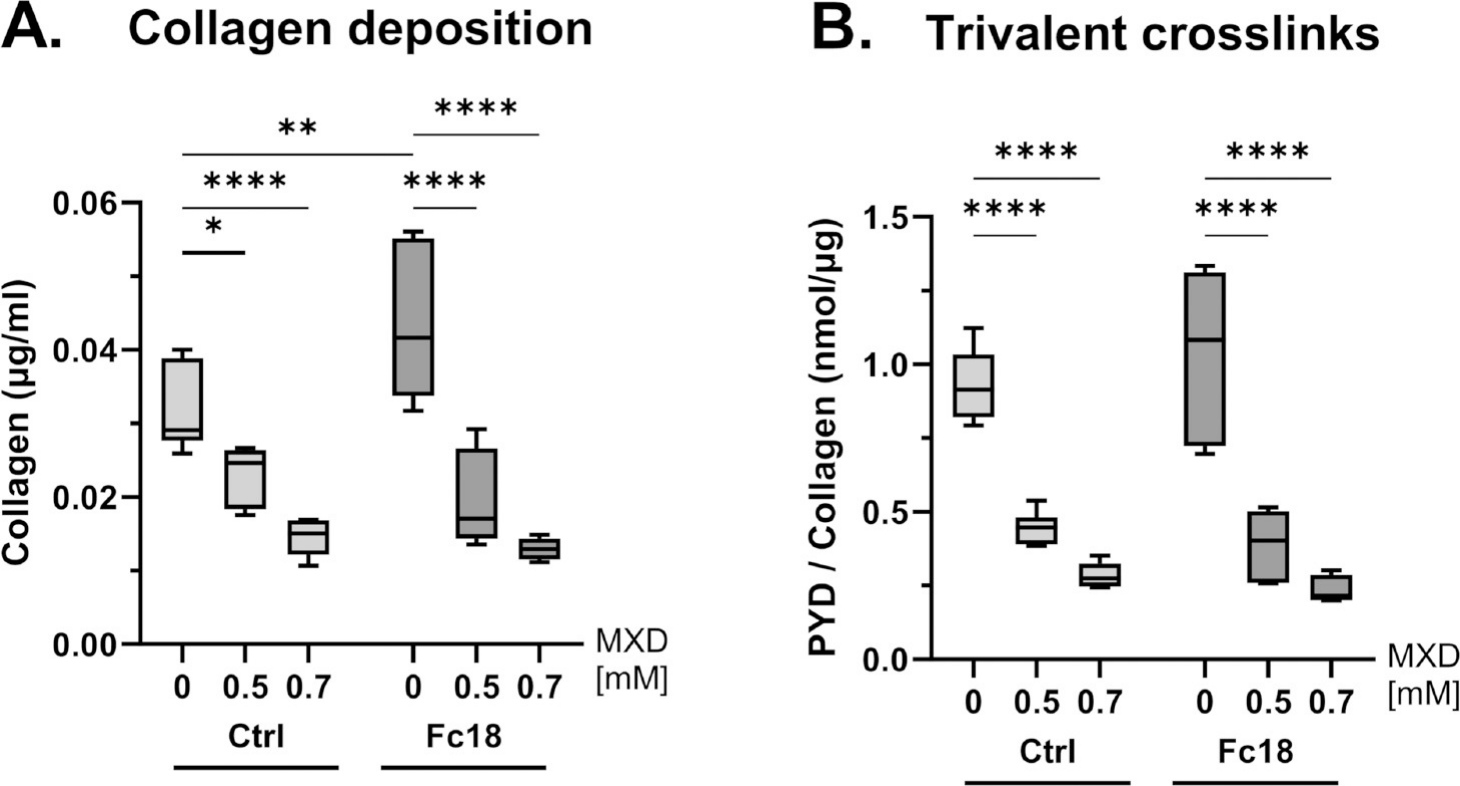
Analysis of collagen matrix deposited by clubfoot-derived (CF-M) cells after 16 days of cultivation in noncrowded Ctrl or macromolecularly crowded Fc18 media with or without minoxidil (MXD) treatment. (A) Long-term collagen deposition was measured by hydroxyproline assay and was normalized to the total protein concentration in the sample. (B) The level of collagen cross-linking was measured as the concentration of pyridinoline and deoxypyridinoline cross-links and is presented relative to the concentration of collagen in the samples. Box plots, Median with IQR (*n* = 6). Two-Way ANOVA, Tukey’s multiple comparisons test (*p < 0.05, **p < 0.01, ***p < 0.001, ****p < 0.0001).

We then studied long-term collagen deposition and cross-linking after 16 days of cultivation in Fc18/Ctrl media with or without MXD treatment. We measured the collagen concentration and the cross-linking following the protocols supplied by kit manufacturers and we optimized the procedures, as described in Capella-Monsonis et al.^61^ First, the total protein content in all samples was estimated by a Pierce BCA assay, and then the hydroxyproline concentration was measured in aliquots of the same samples. We extrapolated the amount of total collagen by multiplying the hydroxyproline content by a factor of 6.6^57^ and we normalized the values to the protein content in the respective samples (Figure 10A). Then we measured the concentration of pyridinoline and deoxypyridinoline trivalent cross-links in the samples, and related it to the amount of collagen (Figure 10B). The total protein values reflected the trend of MXD treatment, causing a reduction in all groups. Normalized collagen deposition was higher in the Fc18 group, reaching a 1.43 times higher median value than in Ctrl (Figure 10A). However, the amount of total collagen per μg of protein in both groups treated with MXD was very similar, with a more significant reduction in the Fc18 groups. The amount of trivalent cross-links in the samples followed the same trend in general. The largest difference between MXD-treated and nontreated groups were in cells grown in Fc18 media. Collagen produced by cells grown in Fc18 without MXD was 1.18-times more cross-linked (Figure 10B), which is below statistical significance level. Nevertheless, the differences in the median values were greater in the Fc18 group, which suggests that the joint effect of Fc18 and MXD on collagen deposition and cross-linking in prolonged cell culture was greater than in the Ctrl group.

## Discussion

The introduction of macromolecular crowding (MMC) in standard *in vitro* cell culture has been proven beneficial, and has been advocated in numerous studies for use in drug screening.^25,33,37,62^ MMC has found application in cancer research,^63^ in studies of cell differentiation into various lineages,^39,41^ in modeling of fibrotic diseases and scarring,^31,33,37,44^ and for the development of ECM-rich cell-derived matrices^24,35^ and cell sheets.^41,45^ However, MMC has never been used with clubfoot primary cells to model the fibrotic conditions of this disease. We took this step to evaluate the effect of MMC on the production of ECM by clubfoot fibroblasts and possibly to harness its benefits in research for clubfoot adjuvant treatment. First, with the addition of MMC, we aimed to create a pseudo-3D biomimetic *in vitro* model of clubfoot fibrosis to set a basis for more effective antifibrotic drug testing. Second, we envisioned combining MMC with a scaffold simulating the stiffness of the original pathological tissue in the future to bring the model even closer to representing the *in vivo* state (i.e., using decellularised clubfoot tissue slices or a decellularised matrix-derived hydrogel prepared from these tissues). Our efforts focus on bridging the gap between standard cell culture and animal models of idiopathic clubfoot.

The work with patient-derived cells is a basic limitation of this study, as primary cells tend to lose their phenotype and, in our case, their pro-fibrotic characteristics during prolonged cultivation. To avoid this, we used clubfoot-isolated cells within their first to third passage, as determined by monitoring cell markers during passaging in our previous study.^22^ However, some interpatient variability in the primary cells is unavoidable. Furthermore, the opportunities to access tissue samples from clubfoot are limited to postrelapse corrective surgeries. Despite the relatively small number of patients included in our study, our set reflected the male:female (2:1) incidence ratio of clubfoot with 5 male and 2 female donors. Idiopathic clubfoot is defined as a disease of multifactorial origin with a possible polygenic nature,^4^ but there is no data available suggesting that the patient’s gender could have any impact on the behavior of isolated cells.

For MMC induction, we selected two different approaches to test: (a) the mix of Ficoll 70 with Ficoll 400 (referred to as Fc) and (b) Polyvinylpyrrolidone 40 (PVP), both of which demonstrated promising results in recent studies with human fibroblasts.^25,32,33,37,38,42^ The ratio of the Ficoll mix that we selected corresponds to the ratio most used in the referenced literature. The range of concentrations that we used in our study to represent the fractional volume occupancy (FVO) of the macromolecules in physiological environments (i.e., 4, 9, 18, 36, and 54% FVO) was inspired by these studies as well, most notably by the study of Rashid et al.^38^ This range follows previously calculated theoretical FVOs, which imitate proteins in interstitial fluid (9% FVO), albumin in bone marrow (corresponds to 18%), blood plasma (45%), and simulate the ECM environment (at 54% FVO). Increasing MMC concentration in cell culture media resulted in a change in its viscosity. However, all of the selected concentrations of both crowding agents were reported to fall within the viscosity of human blood (except for the Fc mix at 54% FVO), with the Fc mix being generally more viscous than PVP at all concentrations.^38^

To facilitate the comparison of our results with the literature, we included normal human dermal fibroblasts (NHDFs) in the initial tests, in addition to cells isolated from clubfoot contracted fibrotic tissue (CF-M) and clubfoot noncontracted tissue (CF-L) from the other side of the foot. The reason for including CF-L as an additional control cell type was the inaccessibility of a more accurate clubfoot control, such as samples from healthy pediatric patients or from patients after successful clubfoot therapy (nonrelapsed). Samples from the lateral side of the foot represent the most feasible option for observing the differences and making comparisons with the contracted medial clubfoot tissue and, in relation, M/L-tissue-derived cells.^19^

All cell types followed a similar trend in their proliferation and metabolic activity in reaction to the tested concentrations of crowding agents (MMCs). In particular, both of our control cell types (NHDFs, CF-L) exhibited a significant decrease (in Fc36, Fc54, PVP36) or even cessation of proliferation (in PVP54), and reduction of metabolic activity when cultivated in a highly crowded environment. This hindrance was presumably due to overcrowding, which caused constrained diffusion of molecules in the media.^64^ The effect was stronger in PVP than in the Fc mix, likely because of the unequal sizes of the hydrodynamic radii (*R*_H_) of the molecules used for crowding (*R*_H_: PVP 40 kDa = 5.1 nm; Fc 70 kDa = 4 nm, Fc 400 kDa = 8 nm),^58,65^ and despite the fact that the reported viscosity of the Fc mix at 36 and 54% FVO is higher than the viscosity of PVP alone.^38^ The hydrodynamic radius influences the volume that the macromolecule excludes from the solution and by extension the diffusion speed of solutes. The difference in conformation of the crowding agents can also play a role. Ficoll is near-spherical in the solution, while PVP adopts random, nonspherical conformations.^66^ The CF-M cells from the clubfoot fibrotic tissue were less prone to overcrowding and they continued to proliferate, albeit more slowly. CF-M cell generally have a longer doubling time, slower proliferation under MMC, and lower metabolic activity during the early stage of the cell culture (day 4) compared to other tested cell types. Due to their slower proliferation rate, these cells probably have lower metabolic requirements, which can facilitate their survival even in highly crowded media. Despite the significant decrease in proliferation, our cells did not die in the high FVO Ficoll mix, as did the fibroblasts in the study of Rashid et al.^38^

When investigating the deposition of extracellular collagen, we have observed an increase in almost all MMC concentrations with all cell types. However, the CF-M cells again showed to be less reactive, or rather the MMC did not elicit such a substantial difference in collagen deposition from their untreated Ctrl as in other cell types. CF-L and NHDFs exhibited very similar deposition profiles, with comparable extracellular collagen deposition levels in Fc4, Fc9, Fc36, Fc54, PVP18, and PVP36, despite the different cell numbers. As previously described, collagen produced in the cultivation environment continues to self-associate even in cell-free systems, which is further promoted by MMC.^27^ Thus, we can presume that the collagen continued to assemble, but as a result, a rather granular form of deposition was observed in groups where the viability of the cells was affected. A similar effect was seen after 48 h of rapid collagen deposition in cell culture with 500 kDa dextran sulfate at 5% FVO by Chen et al.,^29^ and also by Puerta Cavanzo et al.^33^ after 96 h in the same setup. However, cell proliferation did not seem to be inhibited. The study of Rashid et al.,^38^ who were the only group who also compared high concentrations of Ficoll 70 and Ficoll 400 mixed crowding, did not report such a morphological difference in collagen deposited by NHDFs at 36% or at 54% FVO.

We observed the highest peak of MMC concentration efficacy to stimulate collagen deposition in the Fc mix at 18% FVO, which resulted in approximately 4 times more collagen than in the Ctrl groups of CF-L and NHDF cells. Meanwhile, in CF-M the mean difference was only 2.4 times higher relative to Ctrl. The deposition by CF-M under other MMC concentrations also expressed more variation from the trend seen with other cell types. We speculate that this is due to CF-M naturally producing more collagen, which can contribute to crowding of the media. The excluded volume effect of MMC, which increases procollagen processing and collagen deposition, is not as pronounced in CF-M culture as in our control cells. As a result, the difference between collagen deposited in Ctrl and MMC environments is smaller. We did not compare the amount of collagen deposited by the three cell types quantitatively at this point, because we were more interested in the overall differences in the effect of MMC concentrations on clubfoot cells. Other studies consistently present 18% FVO and sometimes 9% FVO of the Fc mix as the best-performing concentration of this crowding agent for ECM deposition fibroblasts,^29,33,38^ which is in agreement with our results. We further supported this conclusion with an analysis of the SHG signal. Among the MMC concentrations that stimulated collagen deposition the best without negatively affecting cell proliferation, we found that only Fc18 significantly accelerated the supramolecular assembly of collagen fibers in all cell types. SHG signals of comparable strength were detected in the culture of CF-M fibroblasts without MMC, usually after 3 weeks.^22^

The standard long culture times eventually enable the cells to self-crowd the media and deposit a comparable amount of collagen,^31^ which indicates that MMC is most effective in early cell culture stages. Our analysis of collagen by fractions following the stages of its biosynthesis^61^ showed that Fc18 also works efficiently during cell culture prolonged to 16 days. A significantly higher amount of collagen secreted by CF-M cells was found in the media supernatant fraction in the Ctrl group. However, it should be noted that most of this water-soluble collagen is taken away during media changes.^58^ Recently deposited collagen in the cell layer, represented by a fraction soluble by pepsin and acetic acid digestion, was also higher in the Ctrl group. At the same time, the insoluble fraction, representing heavily cross-linked collagen, prevailed by far in the Fc18 group, as did the total amount of deposited collagen measured in the cell layer. Macromolecular crowding increases the relative density of procollagen, as well as other peptides produced by the cells such as enzymes.^31^ Enzymes, which are more readily available, can act before they are inactivated or their substrate is dissolved. These enzymes include procollagen N- and C-proteinases, which cleave the propeptide ends of the procollagen molecules, contributing to the decrease of their solubility and initiating collagen fibril formation. The collagen cross-linking enzymes then stabilize the collagen fiber. The conversion from soluble to insoluble collagen is, therefore, continuously promoted by the MMC (Fc18) environment, even after 16 days in culture. Prolonged cultivation in an MMC environment is useful for generating more ECM, measuring biomarkers associated with ECM production over time, and modulating collagen cross-linking.^37^

As a final evaluation of the environment, we examined whether the change from standard culture media to MMC media elicited changes in cellular gene expression. CF-M cells from fibrotic clubfoot tissue showed an increase in fibrosis-associated markers under the influence of an environment crowded with Fc18. After 24 h of supplementation with Fc18 media, the cells exhibited upregulated expression of genes encoding enzymes catalyzing collagen cross-linking—lysyl hydroxylases (LH2, LH3; genes *PLOD2, PLOD3*), pro-fibrotic cytokine TGF-β 1 (*TGFB1*), myofibroblast activation marker α-smooth muscle actin (*ACTA2*), extracellular matrix protein fibronectin (*FN1*), type VI collagen (*COL6A3*), and matrix metalloproteinases (*MMP2, MMP9*). There was no difference in the expression of genes for other enzymes involved in the formation of collagen cross-links, e.g., lysyl oxidase (*LOX*) and prolyl-4-hydroxylase (*P4THM*), or ECM proteins type I and III collagens (*COL1A1, COL1A2, COL3A1*).

The expression profile induced in CF-M cells after cultivation in the MMC (Fc18) environment shares a marked similarity with clubfoot fibrotic tissue. According to our previous research,^19^ both histological staining and gene expression analysis of the fibrotic medial side tissue revealed an increase in TGF-β 1, α-smooth muscle actin, and fibronectin. MMP2 and MMP9 were also upregulated in stained histological sections when compared to the control tissue from the lateral side of clubfoot. At the same time, no increase in gene expression of MMPs was detected. Clubfoot medial side tissue contained a higher accumulation of ECM proteins collagen type I,^18^ type III and type VI than the control tissue, which is in accordance with proteomic data in Eckhardt et al.^17^ Collagen-modifying enzymes lysyl oxidase^19^ and lysyl hydroxylases (except LH1) were also upregulated in the tissues of relapsed clubfoot. In relation to these data, the higher collagen content with a higher level of trivalent pyridinium cross-links found in clubfoot fibrotic tissue has a direct impact through its more difficult degradation,^18^ and influences its biomechanical characteristics such as a higher Young’s elastic modulus.^20^

Although some changes may be tissue-specific, rich representation of ECM proteins like collagens and fibronectin in fibrotic tissues and upregulation of markers such as TGF-β, α-smooth muscle actin, and increased cross-linking are commonly associated with fibrogenesis. MMPs play a multifaceted role in fibrosis; they not only serve as a collagen-degrading enzyme but are generally associated with cell differentiation, ECM maturation and remodeling.^67^ An increase in MMP2 and 9 activity as a function of enhanced ECM deposition stimulated via an MMC environment with carrageenan was reported by Cigognini et al.^39^

*In vitro* fibrotic models are often based on normal healthy cells with supplementation of pro-fibrotic stimuli^33,36,37^ or on cells isolated from pathological tissues with pro-fibrotic phenotype,^18,22,49^ cultivated in standard culture conditions,^49^ in macromolecularly crowded environments,^33,36,37^ or using a combination of these approaches. In these studies, the models were used to screen various drugs with antifibrotic potential (i.e., pirfenidone, nintedanib, omipalisib, minoxidil, BAPN, and ALK5i). Research carried out by Puerta Cavanzo et al.^33^ compared the gene expression of nonstimulated normal human dermal fibroblasts in an MMC environment using an array of various crowding agents. Each tested crowder type had a notably different effect on gene expression after two and 4 days of culture. However, the phenotypic drift that they observed was in the form of downregulation of all genes of interest (*COL1A1, COL1A3, FN1, PLOD2, ACTA2, MMP1*), and occurred basically in all tested MMC environments (including Ficoll mix). They therefore concluded that an external pro-fibrotic stimulus is needed for this cell type, such as TGF-β 1, to continue with antifibrotic drug screening. Simulation of a fibrotic response in normal human lung fibroblasts cultivated in Ficoll mix with TGF-β 1 by Rønnow et al.^37^ increased the synthesis of collagen type I, III, and VI, fibronectin and α-smooth muscle actin. Similarly, Juhl et al.^36^ described different effects on gene expression and ECM production induced by pro-fibrotic stimulation of normal human dermal fibroblasts cultivated in MMC (Ficoll mix) with TGF-β 1, PDGF, or IL-6. TGF-β 1 elicited a typical pro-fibrotic response in healthy cells by stimulating gene expression of various ECM proteins (fibronectin, collagen type I, III, VI, and also type IV and V), α-smooth muscle actin and TGF-β 1 itself. They chose this approach due to limited access to pathological cells from patients with systemic sclerosis or pulmonary fibrosis, which are known to behave differently.^36^ In contrast, clubfoot-derived pathological cells (CF-M) expressed a similar expression profile already after short exposition to an MMC environment even without TGF-β 1 supplementation. Given the general similarities in fibrotic processes, it is likely that MMC models consisting of normal fibroblasts with an adequately selected crowding agent and pro-fibrotic stimuli that elicit the same response or a similar response as clubfoot-derived cells, could produce results that are translatable to relapsed clubfoot and *vice versa*.

In the next part of our study, we employed the established MMC environment of Fc18 as a biomimetic model of clubfoot fibrosis to evaluate the effect of minoxidil (MXD). Minoxidil has been approved by the U.S. Federal Drug Administration (FDA) for hypertension and hair loss treatment (for a review, see Gupta et al.^68^). Due to the function of MXD as a nonspecific inhibitor of lysyl hydroxylase enzymes that catalyzes the formation of pyridinoline cross-links in collagen, it was also explored as a promising antifibrotic agent. Researchers tested its properties in cultures of human fibroblasts from normal dermis or keloid and in keratinocytes,^49,69,70^ in primary fibroblastic synovial cells,^71^ and after administration to mice to alleviate pulmonary fibrosis,^60^ with very satisfactory results. Previous results of our group had shown the effect of MXD for the first time in clubfoot-derived fibroblasts.^22^ Here, we correlate and expand on these results with more in-depth analyses to explore and compare the effect of MXD in a standard cell culture and in an MMC environment.

Following the dose-dependent inhibitory effect of MXD on cell proliferation across a range of concentrations (0–2 mM) in both Ctrl and Fc18 media, we observed a moderately positive effect of Fc18 on cell proliferation during a 164-h experiment. In both cases, we determined 0.75 mM of MXD as the highest safe concentration for CF-M cells. At this concentration of MXD, the cell count measured at the end of the experiment had dropped to approximately 40–50% in comparison to the cells growing in both media without MXD. The proliferation of CF-M cultivated with 0.5 and 0.75 mM slows down significantly after day 4 of culture, but this did not cause direct cytotoxicity.^22^ The same effect was reported for dermal fibroblasts.^69^ Limiting the proliferation of cells in a fibrotic environment could be an added benefit to the antifibrotic action of the tested drug. We therefore reduced the tested concentrations for further analyses accordingly.

The higher initial seeding density of cells in the experiments regarding collagen production erased practically any inhibitory effect of 0.25 mM MXD on cell proliferation in both Ctrl and Fc18 media. The inhibition was attenuated in the case of 0.5 mM MXD, but stayed the same as before when 0.75 mM MXD was added. Since the cell proliferation was similar in the MXD group in both media environments, we evaluated the collagen type I deposition per cell this time. The collagen deposition in the Ctrl media declined with gradually increasing MXD concentrations, following the same trend in the percentage decrease in collagen deposition as in our previous study.^22^ In the Fc18 media, however, the gradual decrease in collagen was less pronounced. When we compared the mean percentages of each group, the slope was very similar to our previous results with the same MXD concentrations in the Ctrl media after 3 weeks of cultivation in the same study. Such similarities indicate that it is effective to accelerate collagen deposition via the Fc18 environment. Concentrations of 0.5 and 0.75 mM of MXD significantly reduced the collagen deposition in both culture environments.

Similarly as in the study of Zuurmond et al.,^49^ the gene expression analysis after 24 h of MXD supplementation to the cells revealed a significant decrease in all three isoforms of lysyl hydroxylases (LH1, LH2, LH3) in the Ctrl and Fc18 media. Moreover, MXD entirely prevented the increase in LH expression that we had observed in cells under the influence of the base Fc18 media. The expression of LHs in MXD-treated Fc18 was inhibited to the same extent as (in the case of LH1, LH3) or significantly more than (in the case of LH2) the expression in MXD-treated Ctrl. At the same time, we detected no significant changes in gene expression for procollagen chains (*COL1A1*, *COL1A2*). A study by Shao et al.^60^ detected an increase in the expression of genes for LH1, LH2, and LH3 in the serum of patients with idiopathic pulmonary fibrosis, and also as in the lung tissue of the mice fibrosis model induced by bleomycin in comparison with healthy animals. After they treated these mice with MXD, the *in vivo* expression levels were still significantly higher than in the control. In the studies of Zuurmond et al.^49^ and Sarkovich et al.,^71^ and also in a previous study of our group,^22^ the addition of MXD alone did not affect the expression of procollagen chains at any time interval. Unfortunately, Shao et al.^60^ did not measure changes in any reported genes outside the bleomycin group. The main inhibitory effect of MXD on collagen deposition is thus realized through the downregulation of collagen-modifying enzymes, e.g., lysyl hydroxylases, and by limiting the amount of hydroxylysine for hydroxyallysine cross-link formation in collagen.^53,72^

The effectiveness of MXD in reducing the hydroxyproline content and the pyridinium cross-links (pyridinoline, deoxypyridinoline) that are elevated in models of fibrotic diseases were documented in studies with the use of MMC environments^60^ and without the use of MMC environments.^71^ The long-term impact on the extracellular matrix deposited by CF-M cells cultured under MXD influence for 16 days was amplified in our model environment, represented by crowded Fc18 media. The combined effect of MXD and Fc18 produced significantly greater inhibition of collagen deposition and greater inhibition of collagen cross-linking. Although the potential of MXD as an antifibrotic agent has been recognized, its influence on reducing each individual isoform of lysyl hydroxylase differs.^49,60^ Considering the predominant role of hydroxyallysine cross-links mediated by lysyl hydroxylase 2 in fibrosis, the search for a structural analog or derivative that would serve as its targeted inhibitor^73,74^ is an important topic for future studies.

## Conclusions

Our study has presented a unique analysis of an *in vitro* fibrosis model combining cells derived from the contracted tissues of relapsed clubfoot patients with a biomimetic cultivation environment induced by macromolecular crowding (MMC). Mixed MMC of differentially sized Ficolls applied at 18% FVO provided the most favorable conditions of all tested variants, outperforming Polyvinylpyrrolidone. The environment induced by Ficoll stimulated the expression of fibrosis-related markers in clubfoot-derived cells, which is consistent with the results of the original contracted tissue analysis. It also promoted the *in vitro* conversion rate of soluble collagen into insoluble collagen, while markedly enhancing collagen deposition and supramolecular assembly during short-term experiments. A prolonged cultivation mode enabled the formation of a thick layer of mature extracellular matrix, which we used to evaluate minoxidil as a potential antifibrotic agent. The application of minoxidil confirmed a dose-dependent effect on the reduction of collagen deposition, and revealed a marked reduction of collagen cross-linking in the extracellular matrix of clubfoot-derived cells. Our model environment enhanced both effects.

Our findings set a basis for broadening research on clubfoot by (a) defining the parameters of clubfoot fibrosis and (b) setting a simple pseudo-3D *in vitro* model of clubfoot fibrosis. The parameters of this model are transferable and can be simulated even without access to clubfoot-derived cells, e.g., with healthy fibroblasts and appropriate pro-fibrotic stimuli in a crowded cell culture environment for the purpose of drug testing.

A macromolecularly crowded cell culture brings the benefit of a biomimetic environment and an option of short cultivation periods, creating a high-throughput platform for drug screening and for early drug development studies. The application of our findings in a standard cell culture or in combination with 3D biofabricated scaffolds or decellularised tissue slices in the future can facilitate the search for potential treatments to reverse local fibrosis, including less research-exposed conditions such as clubfoot.

## Data Availability

The data supporting the conclusions of this study are available within this article and its Supporting Information file or from the corresponding author upon reasonable request.

## Supporting Information Available

Additional experimental data, table and figures describing tissue sample donors, methodology and results of proliferation and cytotoxicity assays (PDF)
