# Supplementary material for: Harnessing the Biomimetic Effect of Macromolecular Crowding in the Cell-Derived Model of Clubfoot Fibrosis
Source: Biomacromolecules. 2024 Aug 30;25(10):6485–502. doi: 10.1021/acs.biomac.4c00653 (PMC11480992; doi:10.1021/acs.biomac.4c00653)
Supplement: Supplementary file 1 — bm4c00653_si_001.pdf [file bm4c00653_si_001.pdf]

Supporting information for

Harnessing the biomimetic effect of macromolecular crowding in the cell-derived model of clubfoot fibrosis

Martina Doubková [1,2,], Jarmila Knitlová [1,3], David Vondrášek [4], Adam Eckhardt [5], Tomáš Novotný [6,7], Martin Ošťádal [8], Elena Filová [1], Lucie Bačáková [1]*

[1] Laboratory of Biomaterials and Tissue Engineering, Institute of Physiology of the Czech Academy of Sciences, Videnska 1083, 142 00 Prague 4, Czech Republic

[2] Second Faculty of Medicine, Charles University, V Uvalu 84, 150 06 Prague 5, Czech Republic

[3] Faculty of Science, Charles University, Albertov 6, 128 00 Prague 2, Czech Republic

[4] Laboratory of Biomathematics, Institute of Physiology of the Czech Academy of Sciences, Videnska 1083, 142 00 Prague 4, Czech Republic

[5] Laboratory of Translational Metabolism, Institute of Physiology of the Czech Academy of Sciences, Videnska 1083, 142 00 Prague 4, Czech Republic

[6] Department of Orthopaedics, Masaryk Hospital, Socialni Pece 3316/12A, 401 13 Usti nad Labem, Czech Republic

[7] Department of Histology and Embryology, Second Faculty of Medicine, Charles University, V Uvalu 84, 150 06 Prague 5, Czech Republic

[8] Department of Orthopaedics, University Hospital Bulovka, Charles University, Budinova 67/2, 180 81 Prague 8, Czech Republic

* Email: Martina.Doubkova@fgu.cas.cz

Contents

1. Biological samples: Patient tissues 3
Table S1 3
2. Methods: Proliferation and cytotoxicity assays 4
Cell mitochondrial activity 4
ECIS - real-time, label-free cellular analysis............ 4
PicoGreen dsDNA assay 4
3. Results 5
3.1 MMC controls cell proliferation and metabolic activity depending on MMC concentration and cell type sensitivity............ 5
Figure S1 6
3.2 MMC (Fc18) positively influenced the proliferation of clubfoot-derived fibroblasts in the presence of minoxidil 7
Figure S2............ 7

1. Biological samples: Patient tissues

Table S1. Data of 7 patients (tissue sample donors) with relapse of idiopathic congenital clubfoot whose primary cells were used for the present study.

Patient	Gender	Dimeglio clubfoot classification	Patient's age during sample acquisition	Ponseti casting	Type of surgery	Previous surgeries	Clubfoot family anamnesis
1	M	III	84	5	MK	AT+R	N
2	M	IV	66	6	PR	AT	N
3	M	III	58	6	PR	AT	N
4	M	IV	40	6	MK	AT	N
5	M	III	19	5	PR	AT	N
6	F	IV	14	10	MK	AT+R	N
7	F	III	81	6	PR	AT	N
Total 7	5x M 2x F	4x III 3x IV	Mean = 51.7 months (SD = 28.2)	Mean = 6	3x MK 4x PR	2x AT+R 5x AT	0 / 7

Parents of these patients were generally non-compliant with the Ponseti regime, unable to maintain proper treatment by application of an abduction bar or even come to scheduled examinations. Abbreviations: M (male), F (female) AT (Achilles Tendon Tenotomy), AT+R (Achilles Tendon Tenotomy + Retenotomy), MK (McKay), N (No), Y (Yes), PR (Posteromedial Release).

2. Methods: Proliferation and cytotoxicity assays

2.1 Cell mitochondrial activity

The effect of different MMC media on cell metabolic activity was assessed using a resazurin assay. As this assay is based on the activity of mitochondrial enzymes, it also provides indirect information on cell proliferation and viability. Prior to the assay in the chosen time intervals (day 4, 7 and 11 of cultivation), the cells were washed with a fresh culture medium without phenol red supplemented with 10% FBS. Then, a 40 µM resazurin solution (R7017, Sigma-Aldrich, USA) diluted in the above-mentioned medium was added to each well and was incubated for 2.5-4 hours in an incubator (37°C, humidified atmosphere with 8% CO_2), protected from light. The fluorescence of the solution was measured in triplicate aliquots using a Synergy HT microplate reader spectrophotometer (BioTek Instruments, USA) at Ex/Em = 530/590 nm. The results were corrected to the blank control (resazurin solution without cells).

2.2 ECIS - real-time, label-free cellular analysis

The ECIS (Electric Cell-substrate Impedance Sensing) Z-Theta (Applied BioPhysics, USA) machine was used to determine the non-toxic concentration of MXD in media with/without MMC. This instrument is capable of label-free, non-invasive monitoring of cell behaviour in real-time using electric cell-substrate sensing. The changes in cell coverage in terms of their attachment and spreading on the well bottom are reflected by the relative change in resistance over time, which is calculated automatically by the instrument software. For ECIS monitoring, clubfoot cells were seeded in an ECIS 96 well micro titer plate with interdigitated electrodes of 3.92 mm^2 per well (96W20idf PET, Applied BioPhysics, USA) at a density of $3.5x10^3$ cells per well (approx. 10,870 $cells/cm^2$), and in 200 µl of medium supplemented with 20% FBS. The cells were left in the incubator for 30 minutes before the plate was mounted onto the ECIS array to start the monitoring. After 24 hours of monitoring, cells were treated with 0, 0.25, 0.5, 0.75, 1 and 2 mM MXD in media with/without MMC and 10% FBS, and the monitoring continued. After 48 hours the cells were supplied with fresh media and were cultivated for another 72 hours to the next media change, to a total of 160 hours of culture. The measurement was conducted in a multifrequency mode. The native morphology of the cells grown on the plate was inspected under an optical microscope in spaces between the electrodes, and the plate was used for a PicoGreen dsDNA assay.

2.3 PicoGreen dsDNA assay

Cells grown on an well plate during the ECIS real-time measurement were used for further analysis after the end of the experiment to quantify the differences in cell proliferation in the media with/without MMC (Fc18) and MXD. The amount of cellular DNA was determined using a Quant-iT PicoGreen dsDNA assay kit (P7589, Invitrogen, Thermo Fisher Scientific, USA) according to the manufacturer's instructions. The fluorescence of the solution was measured in quadruplicate with a Synergy HT microplate reader spectrophotometer (BioTek Instruments, USA) at Ex/Em = 485/528 nm.

3. Results

3.1 MMC controls cell proliferation and metabolic activity depending on MMC concentration and cell type sensitivity

We investigated the effect of Ficoll 70 kDa + Ficoll 400 kDa mix (Fc) and Polyvinylpyrrolidone 40 kDa (PVP) in a range of concentrations (Fc 4-54, PVP 9-54% FVO v/v in the media) on all three cell types. We seeded primary cell cultures of clubfoot-derived fibroblasts from the fibrotic contracted tissue of relapsed patients (CF-M) and from non-contracted clubfoot tissue from the opposite side of the foot (CF-L) in parallel with normal human dermal fibroblasts (NHDFs).

After 24 hours, we added fresh media with or without MMC. On day 4, we observed the cells in their native state under phase-contrast microscopy (Figure S1A). As a complementary analysis, we measured cell viability/metabolic activity with a resazurin assay on separate 96-well plates on day 4, 7 and 11 to see how the cells react to prolonged exposure to MMC (Figure S1B). We quantified the cell nuclei counts in samples at day 7 (Figure 1A, Figure 2A, Figure 3A in the main text of the article).

All three cell types proliferated well in both tested MMC agents up to 18% FVO. However, concentrations of 36% FVO (Fc36, PVP36) and especially 54% FVO (Fc54, PVP54) appeared to inhibit the proliferation of all cell types. This inhibition was stronger for NHDFs and CF-L cells, where both the proliferation and the viability of cells grown in PVP36 and PVP54 were relatively low. In some experiments, most of the cells in PVP54 assumed a rather circular morphology instead of the typical fibroblast spindle-shaped morphology. They stopped proliferating or even died throughout the experiment. By contrast, this trend was much less pronounced in CF-M cells isolated from fibrotic tissue, which proliferated significantly more slowly but steadily, even in Fc and PVP at high (i.e. 36% and 54%) FVO concentrations, usually reaching confluence on day 7 of culture. The cell density on day 7 was in accordance with the metabolic activity of CF-M cells in these MMC concentrations. Interestingly, on day 7, the Fc at 4-18% FVO significantly enhanced the proliferation of NHDF, but only Fc at 18% significantly increased the proliferation in CF-L and CF-M cells (Figure 1A, Figure 2A, Figure 3A in the main text of the article). It should be noted that the overall faster proliferation of NHDFs compared to CF cells despite the same approximate seeding number is consistent with our experience of NHDF having a shorter population doubling time than our primary CF cells. Both CF-M and CF-L, however, reached higher metabolic activity values compared to NHDFs (Figure S1B).

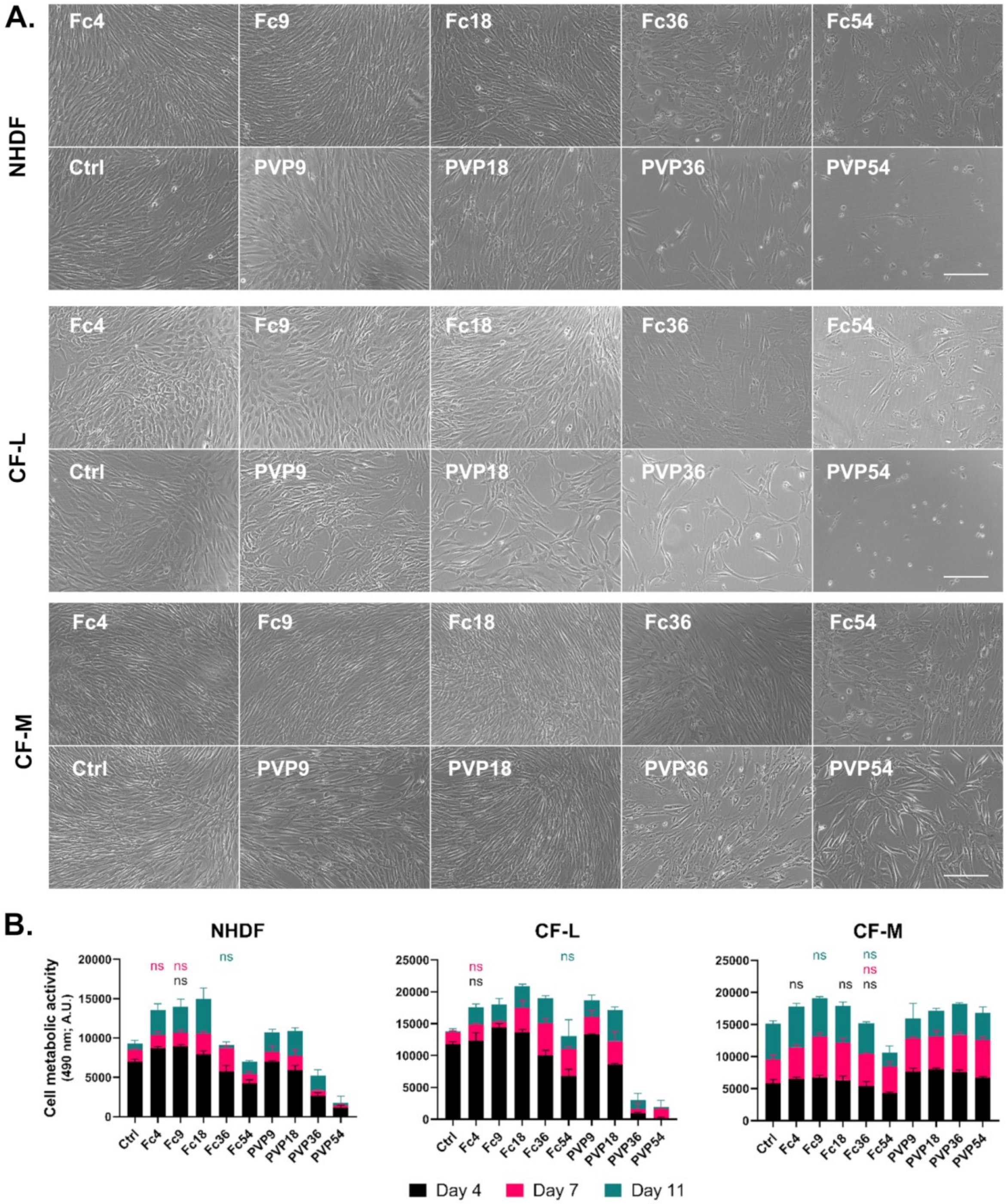

Figure S1. Proliferation and metabolic activity of normal human fibroblasts (NHDF) and clubfoot-derived fibroblasts (CF-L, CF-M) cultivated in various MMC environments. **(A)** The native state of the cells after 4 days of cultivation under phase contrast microscopy. Scale bar = 200 µm. **(B)** Cell metabolic activity on days 4, 7 and 11 after the addition of MMC was measured by resazurin assay. Superimposed bar graphs, mean + SD (n=6). ANOVA, Dunnet's test (vs. Ctrl), $p < 0.05$. Statistical significance vs. Ctrl on respective days. Most values are significant, therefore only non-significant (ns) values are marked in the graphs. NHDF - all significant except PVP9 (day 4), all except Fc36 and PVP9 (day 7), all except Fc36 (day 11). CF-L - all significant except Fc4 (day 4), all except Fc4 (day 7), all except Fc54 (day 11). CF-M - all significant except Fc4, Fc18 and Fc36 (day 4), all except Fc36 (day 7), all except Fc36 and PVP9 (day 11).

3.2 MMC (Fc18) positively influence the proliferation of clubfoot-derived fibroblasts in the presence of minoxidil

We monitored the real-time effect of the absence or presence of minoxidil (MXD) in concentrations of 0, 0.25, 0.50, 0.75, 1, and 2 mM in Ctrl and Fc18 media on clubfoot-derived (CF-M) cells seeded at a density of 10,000 per cm^2. We used the ECIS Theta instrument connected to an array fitted with a 96-well culture plate with golden electrodes on its bottom. The graph in Figure S2A shows the normalised resistance, which indicates the attachment and spreading dynamic of the cells on the well bottom (average of quadruplicate). Subsequently, we measured the amount of cellular DNA in wells of the plate removed from the ECIS instrument after 160 hours of culture. Indeed, we discovered that MXD in concentrations of 1 and 2 mM inhibited cell proliferation significantly in both media types. The amount of cellular dsDNA estimated by PicoGreen assay reached only approximately 29.5% and 10.5%, respectively, to that of a 0 mM MXD group in both Ctrl and Fc18 media (Figure S2B). Generally, there were larger amounts of cells growing in the Fc18 media, i.e. about 30% more than in the Ctrl media. However, the overall reduction in the amounts of cellular DNA at a specific MXD concentration between the two media groups relative to their respective control ranged from 2% to 12%, due to the steeper gradient of the Fc18 graph line. With the exception of the highest concentration of 2 mM, where the values basically matched, we found no other differences between the Ctrl/Fc18 media and the MXD concentrations that were significant at this point. We therefore excluded 1 and 2 mM MXD from further analysis, due to their strong inhibitory effect on cell proliferation.

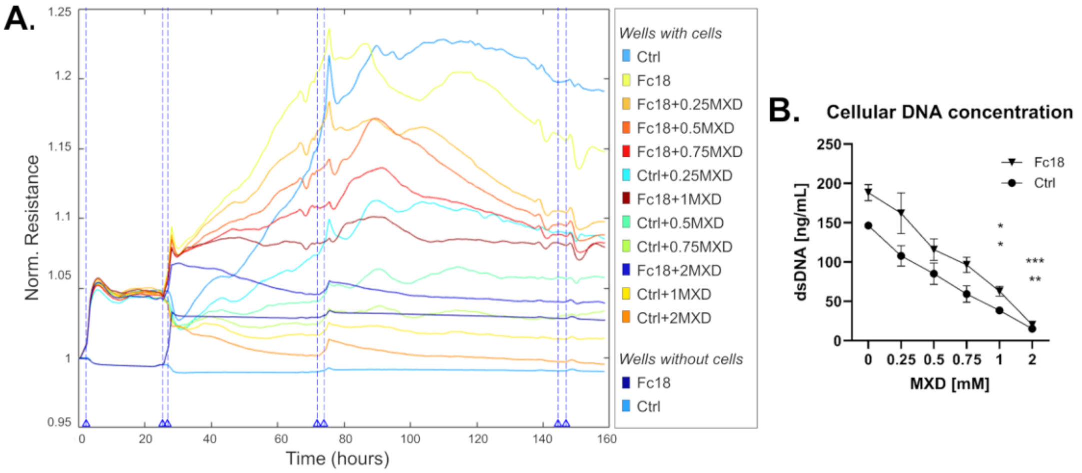

Figure S2. The effect of different concentrations of minoxidil (MXD) of clubfoot-derived cells (CF-M) cultured without (Ctrl) or with (Fc18) macromolecularly crowded media. **(A)** The plot shows the changes in normalised resistance as a function of time as measured during the 160-hour experiment by the ECIS instrument. The mean of 4 wells represents the attachment and spreading dynamic of the cells on the well bottom. Wells without cells show a difference in base measurement for the non-crowded Ctrl or for the macromolecularly crowded Fc18 media only. Vertical blue dashed lines indicate periods where the culture media was changed. The very first dashed line marks the start of the measurement after cell seeding. **(B)** Analysis of the cellular DNA concentration in the wells of the ECIS 96-well plate at the end of the 160-hour experiment. Mean ± SD (n=3-4). Kruskal-Wallis ANOVA, Dunn's test (vs. group respective 0 mM MXD; $*p < 0.05$, $**p < 0.01$, $***p < 0.001$).
